# Extracellular NK histones promote immune cell anti-tumor activity by inducing cell clusters through binding to CD138 receptor

**DOI:** 10.1186/s40425-019-0739-1

**Published:** 2019-10-16

**Authors:** B. Martín-Antonio, G. Suñe, A. Najjar, L. Perez-Amill, A. Antoñana-Vildosola, M. Castella, S. León, M. Velasco-de Andrés, F. Lozano, E. Lozano, C. Bueno, J. M. Estanyol, C. Muñoz-Pinedo, S. N. Robinson, A. Urbano-Ispizua

**Affiliations:** 1 Department of Hematology, Hospital Clinic, IDIBAPS, Carrer Rosselló 149-153, 08036 Barcelona, Spain; 2 Josep Carreras Leukaemia Research Institute, Carrer Rosselló 149-153, 08036 Barcelona, Spain; 3 Department of Pediatrics – Research, The University of Texas M. D. Anderson Cancer Center, Houston, TX USA; 4 Immunoreceptors of the Innate and Adaptive System Group, IDIBAPS, Barcelona, Spain; 5 Josep Carreras Leukemia Research Institute and Cell Therapy Program of the School of Medicine, University of Barcelona, Barcelona, Spain; 6 Department of Immunology, Hospital Clinic of Barcelona, Barcelona, Spain; 7 Department of Biomedical Sciences, School of Medicine, University of Barcelona, Barcelona, Spain; 8 Proteomic department, University of Barcelona, Barcelona, Spain; 9 Cell Death Regulation Group, Oncobell Program, Bellvitge Biomedical Research Institute (IDIBELL), Barcelona, Spain; 10 Department of Stem Cell Transplantation & Cellular Therapy, The University of Texas M. D. Anderson Cancer Center, Houston, TX USA; 11 Department of Hematology, University of Barcelona, Barcelona, Spain

**Keywords:** NK cells, Multiple myeloma, Cell-cell communication, Histones, Immunotherapy

## Abstract

**Background:**

Natural killer (NK) cells are important anti-tumor cells of our innate immune system. Their anti-cancer activity is mediated through interaction of a wide array of activating and inhibitory receptors with their ligands on tumor cells. After activation, NK cells also secrete a variety of pro-inflammatory molecules that contribute to the final immune response by modulating other innate and adaptive immune cells. In this regard, external proteins from NK cell secretome and the mechanisms by which they mediate these responses are poorly defined.

**Methods:**

TRANS-stable-isotope labeling of amino acids in cell culture (TRANS-SILAC) combined with proteomic was undertaken to identify early materials transferred between cord blood-derived NK cells (CB-NK) and multiple myeloma (MM) cells. Further in vitro and in vivo studies with knock-down of histones and CD138, overexpression of histones and addition of exogenous histones were undertaken to confirm TRANS-SILAC results and to determine functional roles of this material transferred.

**Results:**

We describe a novel mechanism by which histones are actively released by NK cells early after contact with MM cells. We show that extracellular histones bind to the heparan sulfate proteoglycan CD138 on the surface of MM cells to promote the creation of immune-tumor cell clusters bringing immune and MM cells into close proximity, and thus facilitating not only NK but also T lymphocyte anti-MM activity.

**Conclusion:**

This study demonstrates a novel immunoregulatory role of NK cells against MM cells mediated by histones, and an additional role of NK cells modulating T lymphocytes activity that will open up new avenues to design future immunotherapy clinical strategies.

## Introduction

Natural killer (NK) cells are important anti-tumor cells of our innate immune system whose anti-tumor properties led to anti-cancer, immune NK cell therapies under development [1]. The majority of clinical studies infusing NK cells worked mostly for acute myeloid leukemias but performed poorly in other malignancies [2, 3], suggesting that a deeper knowledge of NK cells is required to better understand and exploit their anti-tumor activity. In this regard, NK cells present a wide array of activating and inhibitory receptors that interact with their ligands on tumor cells [4]. However, besides these receptor-ligands interactions, a cross-talk among different immune cells, performed by pro-inflammatory molecules secreted by immune cells, contributes to the final immune response [5].

The relevance of this cross-talk between immune cells is observed after microbial infection, where dendritic cells (DCs) activate NK cells through IL15 secretion leading to T cell and monocyte activation [5–7]. The coordination of these immune responses requires the creation of cellular clusters to enable intercellular cross-talk between immune cells [7, 8]. We previously reported the relevance of this cell-cell contact as a mechanism leading to a transmissible cytotoxicity from cord blood derived NK cell (CB-NK) to neighboring multiple myeloma (MM) cells, as CB-NK cytotoxicity is transferred to ‘primary’ MM cells (1°MM) after contact; and afterwards, it is passed from 1°MM to adjacent ‘secondary’ MM cells (2°MM) unexposed to CB-NK [9]. Interestingly, CB-NK perform Granzyme-B and Caspase-3 independent killing of MM cells [9], suggesting the involvement of other proteins in the CB-NK anti-MM activity. Moreover, whereas effector cytokines require hours to be detected, cell cluster formations occur earlier, suggesting that other initiating molecules secreted at early times of cell-cell contact will impact on the final effector response.

These observations led us to hypothesize that novel cytotoxic molecules transferred from CB-NK to MM cells could be involved in the anti-MM CB-NK activity. Therefore, TRANS-stable-isotope labeling of amino acids in cell culture (TRANS-SILAC) [10] combined with proteomic was undertaken to identify early materials transferred between CB-NK and MM. Analysis revealed that histones are actively transferred between CB-NK and MM and also released into the extracellular milieu after co-culturing CB-NK and MM. Released CB-NK histones bind to CD138 in MM cells promoting the formation of CB-NK/MM cell clusters which facilitates NK-MM contact and improves the anti-tumor NK efficacy. Furthermore, NK-histones also promoted the generation of cell clusters between T-cells and MM cells increasing the T cell anti-MM activity and revealing a novel mechanism by which NK enhance the anti-tumor activity of T-lymphocytes.

## Methods

### Cell cultures

NK cells were isolated from CB and PB by magnetic depletion (Miltenyi Biotec). CB-NK expansion was performed during 14 days as previously described [9] using K562-based antigen presenting cells expressing membrane bound IL-21 (“Clone 9.mbIL21”). T cells were isolated from PB by magnetic depletion (Miltenyi Biotec) and expanded during 5 days with Dynabeads® Human T-Activator CD3/CD28 (Thermo-Fisher). IL2 (Proleukin) was added at 100UI/mL every other day. Culture NK and T cell media was comprised of 45% RPMI-1640 (Sigma-aldrich) and 45% Click’s (Irvine Scientific) with 10% AB human serum (Atlanta Biologicals). ARP1 cell line was provided by Multiple Myeloma Research Center (Little Rock, AK). 293 T, K562, U266, RPMI-8226, Ramos and Jurkat cells were obtained from American Type Culture Collection (ATCC, Rockville, MD). K562, ARP1, RPMI, Ramos and Jurkat were cultured in RPMI-1640 with 10% fetal bovine serum (FBS) and U266 with 15% FBS. 293 T cells were cultured in DMEM with 10% FBS. CD138^+^ cells from MM patients were obtained by MACS selection (Miltenyi Biotec).

### TRANS-SILAC proteomics

Was performed culturing cells in their usual media lacking normal L-Arg, L-Leu and L-Lys, and supplemented with their corresponding heavy isotopic AA (hAA). ARP1-MM cells were expanded in this media for 21 days, and CB-NK during the 14 days of the usual CB-NK in vitro expansion. After this period, both cell populations contained > 97% of hAA as determined by liquid Chromatography-Tandem Mass Spectrometry (LC-MS/MS). Percentage of heavy proteins transferred to each cell population was analyzed by LC-MS/MS.

### Cytotoxicity assays

Were performed at 3 h by Europium Release Assays [9], and at longer times by flow cytometry calculating % of remaining live GFP+ tumor cells applying the formula: % of target cell lysis = 100-(% of GFP+ cells at 24-48 h **/** % of GFP+ cells at 0 h).

### In vivo myeloma murine model

NOD/SCID IL-2Rcnull (NSG) mice were irradiated and inoculated i.v. with GFP-Firefly Luciferase-transduced ARP1 cells. Recombinant H2AZ (0.5 mg/kg) was given i.v. on day 1 and day 7. Disease progression was monitored by bioluminescence using a Hamamatsu CDD camera (Hamamatsu Photonics Sistems) following a 100 mL IP injection of D-luciferin (20 mg/mL), and measuring serum kappa light chain levels by ELISA (Bethyl Laboratories). Signal quantitation was performed with ImageJ software.

### Transfer of H2AZ-GFP transfer between cells

Cells were co-cultured staining in blue (CMAC) the cell population of interest. Then, H2AZ-GFP transfer between cells was analyzed by flow cytometry gating on the CMAC+ population and analyzing the % of CMAC+GFP+ cells.

### Supernatant Containing Inflammatory Proteins (SIPs) analysis

To analyze released proteins to the extracellular milieu by each cell population, 30–40 min co-cultures experiments were performed collecting the supernatants and differentiating proteins of each cell population by their previous hAA labeling (Additional file 1: Figure S1D).

### Reagents

Caspase-1 inhibition was achieved with Y-VAD (50 μM) addition. Heparinase III (Sigma-aldrich) treatment (0.01 IU/mL) was used to remove HSGPG from MM cells [11]. Recombinant H2AZ (Merck-Millipore) and H4 (New England Biolabs) were added in cell culture at 2 μM or 0.5 μM depending on the experiment. Heparin (STEMCELL Technologies) was used at 20 IU/mL. DNAse I (D2) (Worthington Biochemical Corporation) was used at 100 IU/mL. Antibodies used were CD138-V421 and CD56-V450 (BD Biosciences), H2AZ, H4, H1.5, Anti-Rabbit IgG-HRP, and Anti-Rabbit IgG-Alexa Fluor-647 (Cell Signaling Technology).

### Cell cluster formation

Area of cell clusters was visualized at different time points by measuring the GFP area from tumor cells using ImageJ software.

### Ethics Statement

Research involving human materials was approved by Ethical Committee of Hospital Clinic, Barcelona. CB units and PB were obtained from healthy donors who gave informed consent.

### Statistical analysis

Mann-Whitney U test was used to analyze comparison between groups. Statistical analyses were performed with SPSS (IBM SPSS v. 23).

LC-MS/MS, confocal fluorescence microscopy, flow cytometry, GFP-fused protein generation, lentivirus production and siRNA transfection are detailed in Additional file 1: Supplementary Methods.

## Results

### CB-NK histones are dynamically transferred from CB-NK to primary MM cells and subsequently to adjacent secondary MM cells

In order to identify cytotoxic CB-NK proteins transferred directly to MM cells (1°MM), and secondarily to neighboring MM cells (2°MM), TRANS-SILAC proteomic was performed to identify acquired proteome for each cell population [10]. Co-culture experiments were limited to 30–40 min to unravel early transferred proteins between live cells responsible for initiating NK cytotoxicity.

**Fig. 1:**
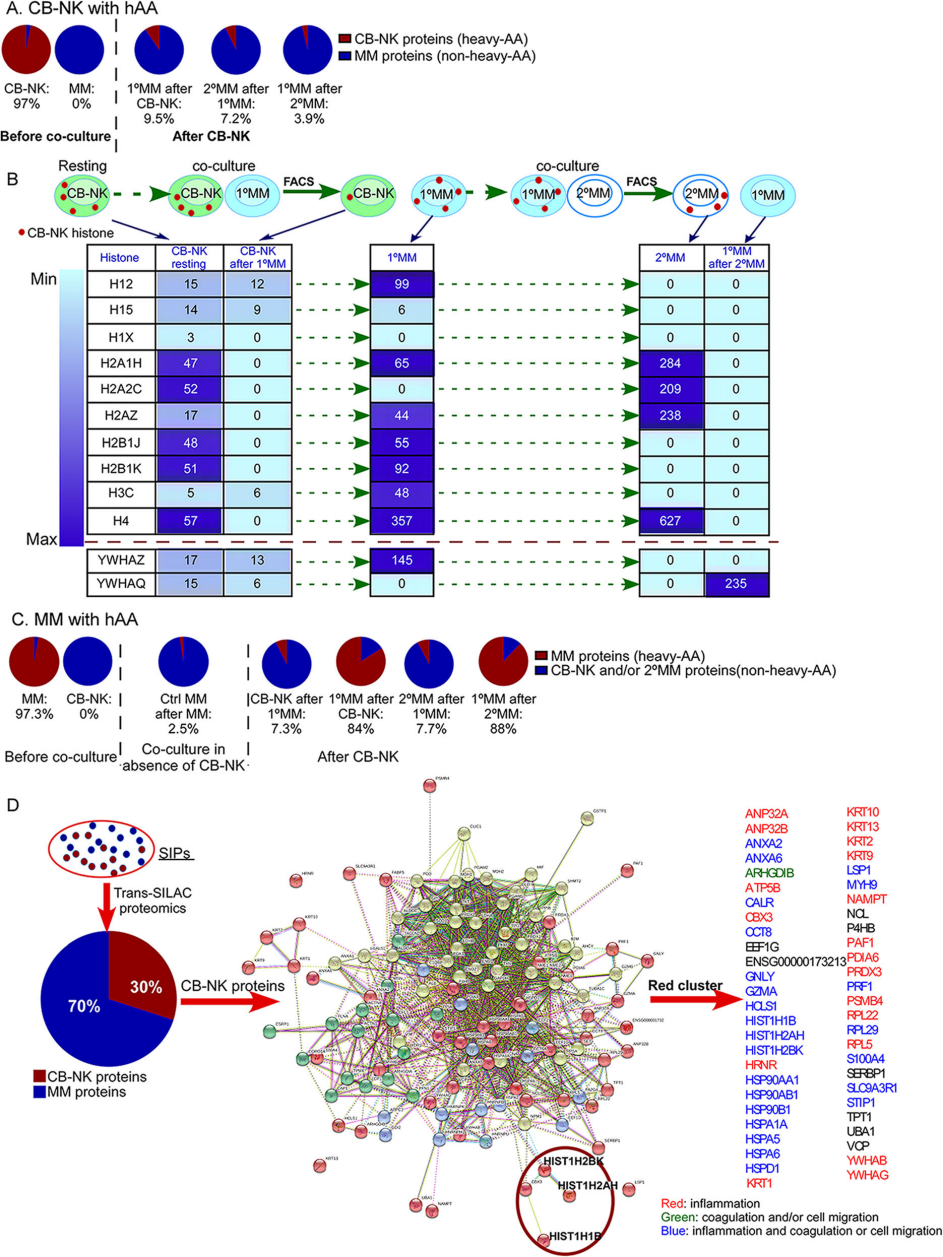
Cord blood derived NK cells (CB-NK) increase cell-cell communication between CB-NK and MM cells, leading to enhanced proteome transfer, including a high number of histones. **a** and **c**: Percentage of heavy-labeled (transferred) proteins from the total cell proteome in each cell population after labeling either CB-NK (**a**) or MM cells (**c**) with heavy amino acids (hAA). Each cell population was obtained after co-culturing and FACS sorting according to Diagram shown in Additional file 1: Figure S1. **b**: Schematic design of the cell populations analyzed which are shown in the Table below to present trafficking of CB-NK histones and other NK proteins through MM cells (Additional file 1: Tables S1, S2 and S3). Numbers in the table indicate number of PSMs (peptide spectral match) detected, indicating the relative abundance of proteins. Scheme shows CB-NK in resting conditions and after co-culture with MM cells (1°MM). Afterwards, 1°MM cells transfer CB-NK histones secondarily to neighboring MM cells (2°MM), with subsequent loss of CB-NK histones in 1°MM cells. **d**. Analysis of released proteins after CB-NK/MM cell co-culture termed Supernatant containing Inflammatory Proteins (SIPs). See diagram shown in Additional file 1: Figure S1D. CB-NK proteins from SIPs are shown in the diagram, and proteins of the red cluster, which includes histones (in a red circle), are detailed. See also Additional file 1: Tables S1-S8 for list of transferred proteins

CB-NK were labeled with heavy amino acids (hAA) to allow for identification of ‘heavy’ CB-NK proteome transferred to 1°MM (labeled with CMAC); and subsequently from 1°MM to 2°MM (unstained) (Additional file 1: Figure S1A). Proteomic data showed that after CB-NK/1°MM co-culture, the 1°MM proteome contained 9.5% of proteins transferred from CB-NK (Fig. 1a) (Additional file 1: Table S1). Then, 1°MM were co-cultured with fresh MM cells to determine CB-NK proteins transferred secondarily between MM cells, revealing 7.2% of secondary transfer of NK proteome from the 1°MM to neighboring 2°MM cells. These proteins were thus originally derived from CB-NK, but via 1°MM (Fig. 1a, Additional file 1: Table S2). As a consequence of this transfer, 1°MM lost part of their labeled, previously acquired CB-NK proteome content that went down from 9.5 to 3.9% (Fig. 1a, Additional file 1: Table S3). These data provided evidence for a primary-direct CB-NK proteome transfer to 1°MM, and a secondary-indirect CB-NK proteome transfer to 2°MM.

Analysis of CB-NK transferred proteins to MM cells showed a high number of CB-NK histones acquired by MM cells. In resting conditions, different histones were present in CB-NK (Fig. 1b: CB-NK resting). However, after co-culturing with 1°MM, CB-NK lost their histone content (Fig. 1b: CB-NK after 1°MM), as indicated by absence of detection of Peptide Spectral Matches (PSMs). Conversely, MM cells underwent an enrichment in these histones (Fig. 1b), suggesting the selectivity of this process. We observed that subsequently, CB-NK histones from 1°MM cells were then transferred to 2°MM cells (Fig. 1b: 2°MM), and as a consequence, the content of labeled, CB-NK histones in 1°MM disappeared (Fig. 1b: 1°MM after 2°MM), suggesting a continuous, dynamic and specific transfer of CB-NK histones between MM cells. Of note, other NK proteins detected in the proteomic data (YWHAZ and YWHAQ) did not show this pattern of continuous transfer observed for histones (Fig. 1b).

### MM cells exposed to CB-NK increase their intercellular communication transferring proteins to CB-NK and to neighboring MM cells

In a complementary approach, MM cells were expanded in vitro with hAA to identify 1°MM proteome transferred to CB-NK and to 2°MM (Additional file 1: Figure S1B). As control, the transmission of MM proteome between MM cells under ‘resting’ conditions (absence of CB-NK) was also investigated (Additional file 1: Figure S1C). After CB-NK exposure, CB-NK received 7.3% of MM proteome (Fig. 1c, Additional file 1: Table S4). Moreover, whereas under ‘resting’ conditions 2.5% of MM proteome was transferred between MM cells (Fig. 1c, Additional file 1: Table S5), after CB-NK, MM proteome transfer between neighboring MM cells increased to 7.7% (Fig. 1c, Additional file 1: Table S6). These experiments suggested that MM cells display a low constitutive transfer of their proteome, which is increased after CB-NK exposure leading to a bidirectional exchange of proteome.

### CB-NK histones are also released into the extracellular milieu after co-culture with MM cells

A third experiment co-culturing hAA-labeled MM cells and CB-NK was performed to analyze released SIPs (Additional file 1: Figure S1D). Proteomic analysis showed that SIPs contained 30% of CB-NK proteins and 70% of proteins from hAA-labeled MM cells (Fig. 1d, Additional file 1: Tables S7 and S8). Clustering analysis of this 30% of CB-NK proteins by using STRING-database showed the presence of histones in this released NK material. Histones are highly involved in inflammation and coagulation mechanisms known as ‘immunothrombosis’ [12]. As shown in Fig. 1d, most of the other NK proteins detected in the same cluster of histones (red cluster) are also involved in inflammation, coagulation and/or cell migration processes.

### CB-NK histones are actively transferred through MM cells

Proteomic data suggested a dynamic movement of CB-NK histones through different MM cells, as 1°MM lost their CB-NK histones by passing them to 2°MM. As histones show antimicrobial [13, 14] and anti-tumor properties [15], further studies were undertaken to confirm their cell-cell transfer and impact on MM cells. Histone variant H2AZ1 (*H2AZ*) was first selected due to the high number of PSMs detected and to its presence in 1°MM and 2°MM cells. In addition, YWHAZ and YWHAQ were also selected for further analysis since these CB-NK proteins were identified either in 1°MM or 2°MM cells and are involved in tumor cell survival [16].

**Fig. 2:**
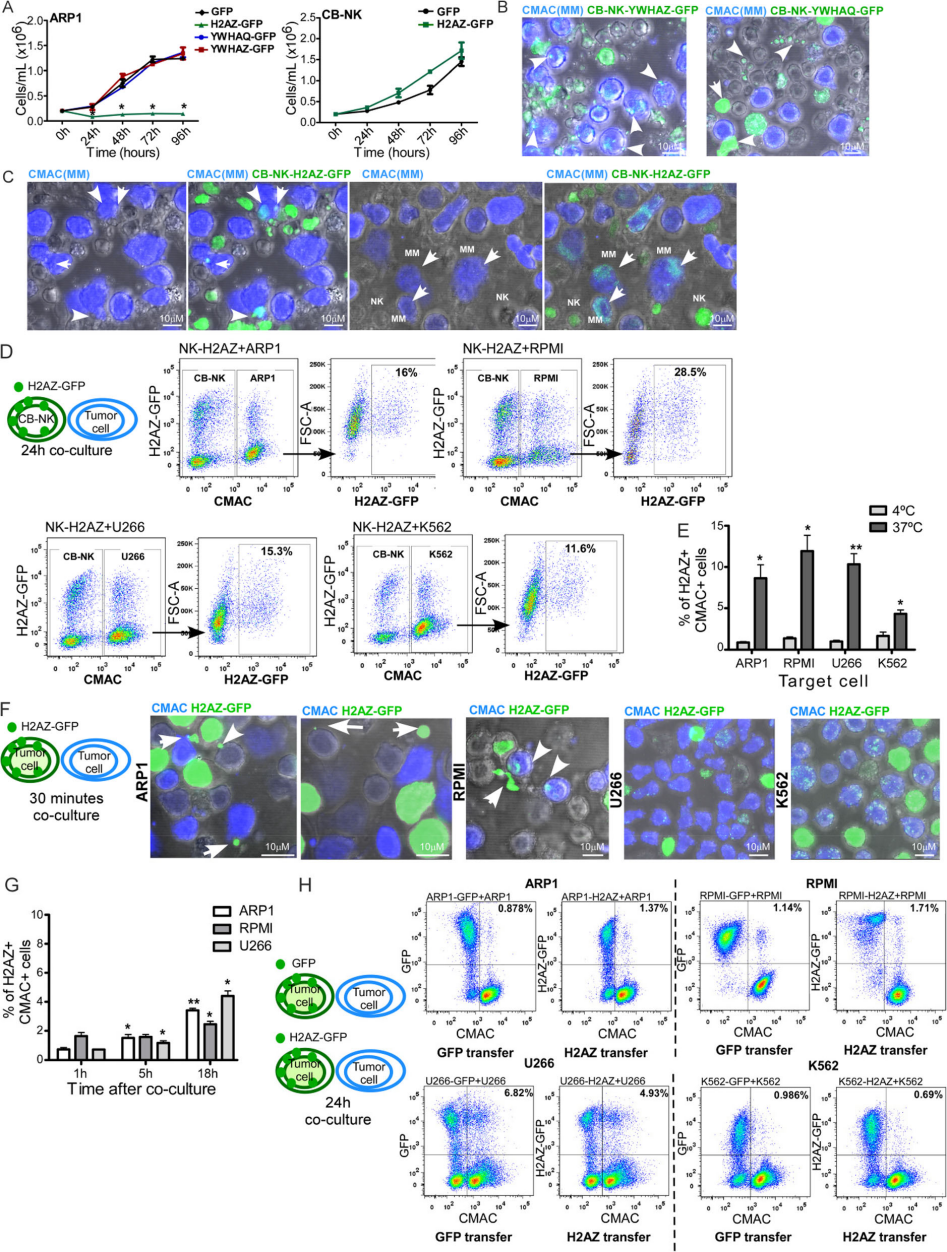
CB-NK histones are actively transferred through MM cells. **a**: ARP1 and CB-NK cell proliferation during four days after over-expression of YWHAQ-GFP, YWHAZ-GFP and H2AZ-GFP vs control, measured by viable cell count. **b**-**c:** Transfer of YWHAZ and YWHAQ (**b**), and H2AZ (**c**) from CB-NK to ARP1 cells. CB-NK transduced with the corresponding protein fused to GFP are co-cultured with ARP1 cells for 30 min. ARP1 cells in blue (CMAC) and CB-NK show in green the corresponding GFP-fused protein. Arrows in **c** indicate H2AZ-vesicles and H2AZ adhered to the surface of MM cells. **d**: Transfer of H2AZ-GFP from CB-NK to MM (ARP1, RPMI and U266) and non-MM K562 cells after 24 h of co-culture. Target cells are shown in blue (CMAC) in plot 1, and plot 2 corresponds to the gate of CMAC+ cells. **e**. Transfer of H2AZ-GFP from CB-NK to MM and non-MM K562 cells after 24 h of co-culture performed in parallel at 37 °C and 4 °C. **f** to **h**: H2AZ can be transferred between tumor cells in a CB-NK independent manner. **f**: Transfer of H2AZ from tumor cells over-expressing H2AZ-GFP to neighboring tumor cells stained in blue (CMAC) after 30 min of co-culture. Arrows indicate H2AZ-vesicles and H2AZ-intercellular structure being transferred to neighboring tumor cells. **g**: H2AZ transfer from MM cells over-expressing H2AZ-GFP to neighboring MM cells in blue (CMAC) at different times (1 h, 5 h, 18 h) of co-culture. Statistical analysis shown is performed for each cell line vs 1 h time point. **h**: Transfer of GFP (plot on the left) and H2AZ-GFP (plot on the right) from MM and non-MM K562 cells over-expressing these proteins to neighboring MM and non-MM K562 cells after 24 h of co-culture. Representative images from at least three independent experiments

H2AZ, YWHAZ and YWHAQ fused to green fluorescent protein (GFP) were overexpressed in MM cells. While YWHAZ-GFP and YWHAQ-GFP over-expression had no effect on the in vitro proliferation of ARP1 cells, H2AZ-GFP over-expression significantly decreased ARP1 proliferation (Fig. 2a), and this inhibitory effect was not observed in CB-NK (Fig. 2a). Moreover, when CB-NK were transduced with these GFP-fused proteins and co-cultured with MM cells for 30 min, transfer of these proteins from CB-NK to MM cells was confirmed. While YWHAQ-GFP and YWHAZ-GFP were transmitted from CB-NK to MM cells in vesicles (Fig. 2b), H2AZ-GFP was transmitted by both vesicles (Fig. 2c) and large intercellular structures co-localizing with DNA (Additional file 1: Figure S2A). Moreover, H2AZ-GFP also appeared to adhere to MM surface (Fig. 2c). In addition, H2AZ-intercellular structures were also detected after co-culturing CB-NK with primary CD138^+^ cells from MM patients (Additional file 1: Figure S2B).

We next analyzed whether CB-NK could transfer H2AZ to other cells besides MM cells. Co-culturing CB-NK-H2AZ-GFP with MM and non-MM (K562) cell lines, showed that a fraction of all tumor cell lines expressed H2AZ-GFP after 24 h (Fig. 2d). To confirm that H2AZ transfer was an active and regulated mechanism, the same experiment was performed in parallel at 4 °C and 37 °C, confirming an increased and active H2AZ transfer from CB-NK to tumor cells at 37 °C, and that the degree of transfer was lower for non-MM K562 (Fig. 2e).

Since H2AZ was transferred from CB-NK to MM cells and then, secondarily between MM cells (Fig. 1b), we next analyzed whether tumor (MM and non-MM K562) cells over-expressing H2AZ could transfer this protein to neighboring tumor cells in the absence of CB-NK. Indeed, transmission of H2AZ-GFP to neighboring tumor cells was observed in the absence of CB-NK (Fig. 2f). The kinetics of H2AZ-GFP transfer between neighboring MM cells was monitored from 1 to 18 h (Fig. 2g) demonstrating that the rate of H2AZ transfer occurred at a much lower rate than in the presence of CB-NK (Fig. 2e) and indicating that histone NK transfer to MM cells is an active process. Different rates of H2AZ-GFP transfer were observed for each cell line. Thus, although for RPMI cells, the initial rate of H2AZ-GFP transfer was relatively high, it increased less with extended time in culture than for ARP1 and U266 cells where the initial rate of H2AZ-GFP transfer was lower (Fig. 2g).

To investigate whether the transfer of materials between cells was unique to toxic proteins, or applicable to all proteins, we compared the transfer kinetics of H2AZ-GFP and GFP. Although the actual rates of transfer and proportion of protein transferred differed between the different cell lines, similar rates of transfer were observed for both molecules (H2AZ-GFP and GFP) for each cell line (Fig. 2h). Altogether, our results indicate that tumor cells transfer proteins between them, and that the presence of CB-NK cells greatly increases the transfer rate.

### Histones are involved in CB-NK anti-MM activity

**Fig. 3:**
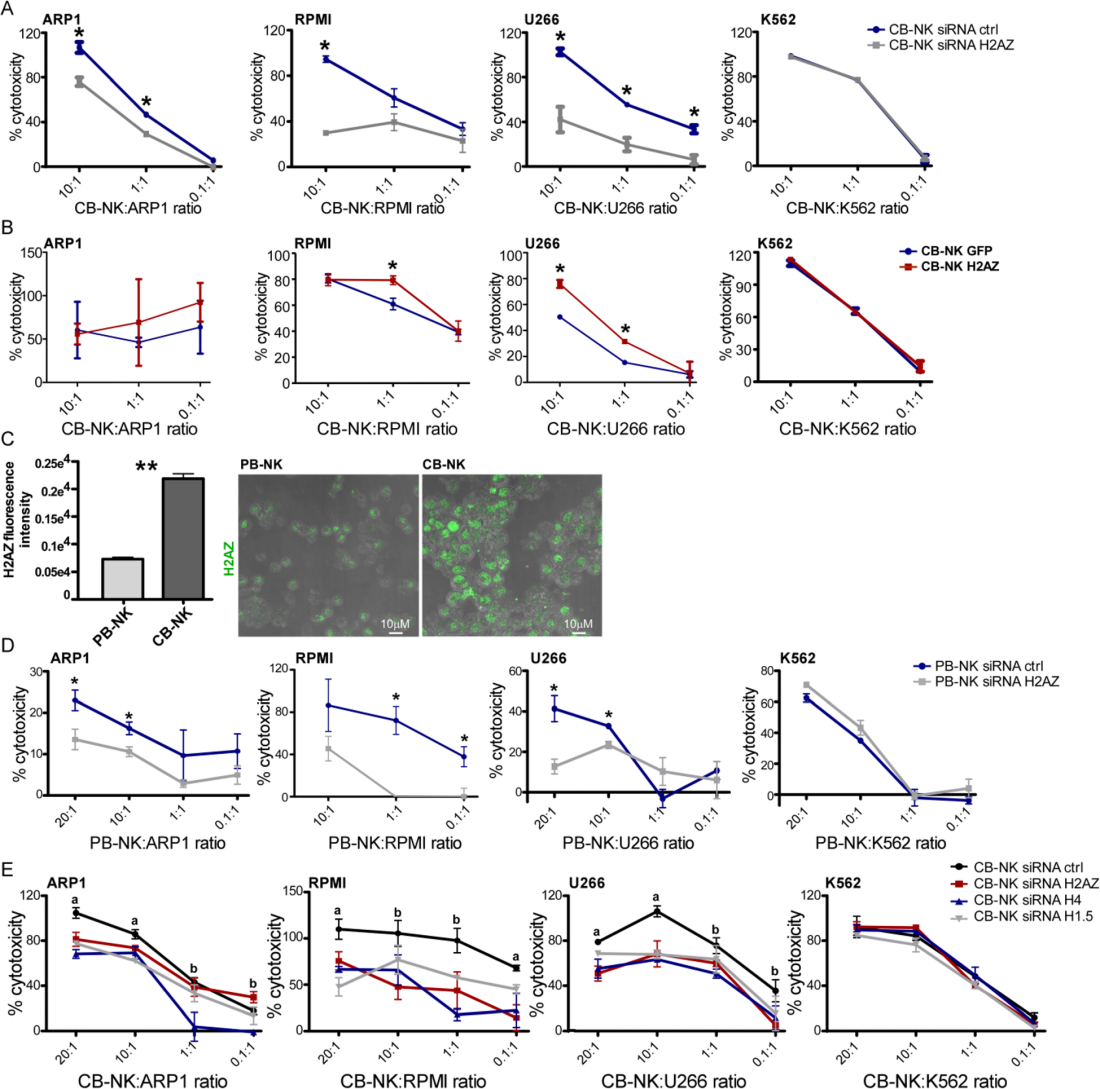
Histones are involved in CB-NK anti-MM activity. **a.** 3 h cytotoxicity assays comparing CB-NK control (CB-NK siRNA ctrl) vs CB-NK with knockdown of H2AZ (CB-NK siRNA H2AZ). **b.** 3 h cytotoxicity assays comparing CB-NK control (CB-NK GFP) vs CB-NK over-expressing H2AZ (CB-NK H2AZ). **c**. H2AZ levels in peripheral blood NK cells (PB-NK) vs CB-NK, analyzed by confocal fluorescence microscopy. Representative image of H2AZ levels is shown on the right. **d.** 3 h cytotoxicity assays comparing PB-NK control (PB-NK siRNA ctrl) vs PB-NK with knockdown of H2AZ (PB-NK siRNA H2AZ). **e**. 3 h cytotoxicity assays comparing CB-NK (CB-NK siRNA ctrl) with CB-NK where histones H2AZ, H4 and H1.5 were knockdown. Assays were performed at least in three independent experiments. **a**: all groups analyzed compared to CB-NK siRNA ctrl are different (*p* < 0.05). **b**: at least one group analyzed compared to CB-NK siRNA ctrl is different (*p* < 0.05). *p < 0.05. ** *p* < 0.001. Efficiency of knockdown of H2AZ was confirmed by Western Blot and by flow cytometry (Additional file 1: Figure S3)

To test whether H2AZ was involved in NK cytotoxicity, we performed knock-down (siRNA) and over-expression of H2AZ in CB-NK. H2AZ knock-down in CB-NK reduced cytotoxicity against MM cells but not against K562 cells (Fig. 3a), suggesting that although H2AZ is transferred from CB-NK to K562 (Fig. 2d) it has no role in the killing of K562. Conversely, H2AZ over-expression in CB-NK increased their cytotoxicity against MM cells in a different degree depending on the MM cell line (Fig. 3b). No effect was detected for K562, consistent with previous studies reporting that NK-cytotoxicity against K562 is mainly mediated through Granzyme B and Caspase-3 [9].

The role of H2AZ in the killing of MM cells by NK was further confirmed by the assessment of peripheral blood (PB)-NK. Although PB-NK expressed lower H2AZ levels than CB-NK (Fig. 3c), H2AZ knock-down reduced PB-NK cytotoxicity against MM cells, a finding not observed against K562 (Fig. 3d), confirming also H2AZ involvement in PB-NK anti-MM activity.

Finally, the impact of other histones (H4 and H1.5) in CB-NK cytotoxicity was also assessed. Individual knock-down of H2AZ, H4 and H1.5 in CB-NK (Additional file 1: Figure S3) decreased CB-NK cytotoxicity against MM cells, and not against K562 cells (Fig. 3e), confirming that, as suggested in the proteomic data, histones are involved in CB-NK anti-MM activity.

### CB-NK and histones promote pyroptosis with in vivo MM cell death and concomitant inflammation

**Fig. 4:**
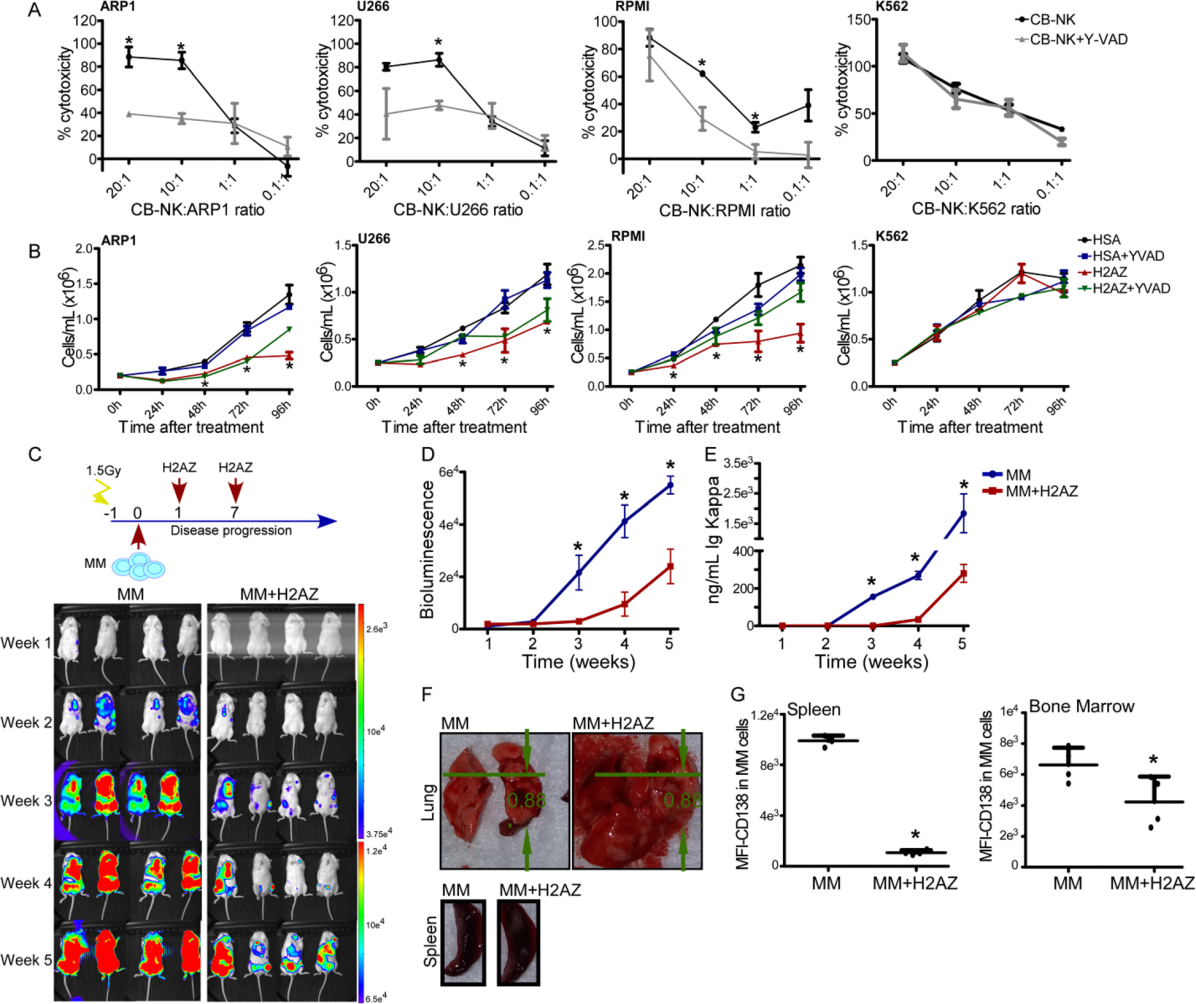
CB-NK and histones promote pyroptosis with in vivo MM cell death and concomitant inflammation. **a**. 3 h cytotoxicity assays of CB-NK against MM and non-MM K562 cells, adding Caspase-1 inhibitor to analyze the impact on pyroptotic cell death. **b**. Impact of recombinant H2AZ on viability of MM and non-MM K562 cells. HSA: Human Serum Albumin (2 μM) was added as protein control in parallel with H2AZ (2 μM). Y-VAD was added to analyze the impact on H2AZ effect. Cell proliferation was measured by viable cell count. **c** to **g**: Anti-MM and pro-inflammatory in vivo activity of H2AZ. NSG mice received ARP1 cells and were treated with recombinant H2AZ. Weekly bioluminescence (**c** and **d**) images and kappa ELISA light chains measurements (**e**) were performed. **f**. Lung and tissues of mice untreated (MM) or treated with H2AZ (MM + H2AZ). **g**: CD138 expression in MM cells of mice tissues. *p < 0.05

Extracellular histones are highly pro-inflammatory and activate the inflammasome leading to pyroptosis, an inflammatory form of cell death caspase-1-dependent [13, 17, 18]. Since NK cells show pro-inflammatory activity [19], and we had previously discarded apoptosis as the form of cytotoxicity [9], we hypothesized that CB-NK-associated histones might induce pyroptosis to kill MM cells. Indeed, we found that inhibition of Caspase-1 decreased CB-NK anti-MM activity, an effect not detected in K562 cells (Fig. 4a). Importantly, addition of recombinant H2AZ induced anti-MM activity (Fig. 4b), a finding also observed for recombinant H4 (Additional file 1: Figure S4). In these experimental conditions, caspase-1 inhibition also reduced H2AZ-mediated cytotoxicity (Fig. 4b).

To assess the anti-MM and inflammatory role for histones in vivo, NSG-mice receiving ARP1 and treated with recombinant H2AZ showed that administration of H2AZ was associated with a remarkable delay in the progression of MM (Fig. 4c-e). However, abnormally enlarged lung and spleen tissues were observed, which could result from inflammatory damage induced by H2AZ (Fig. 4f). Interestingly, the phenotype of MM cells in bone marrow and spleen from, H2AZ-treated mice presented decreased intensity of CD138 (Fig. 4g), a marker highly expressed on MM cells.

### NK-histones specifically bind to CD138 on MM cell surface

**Fig. 5:**
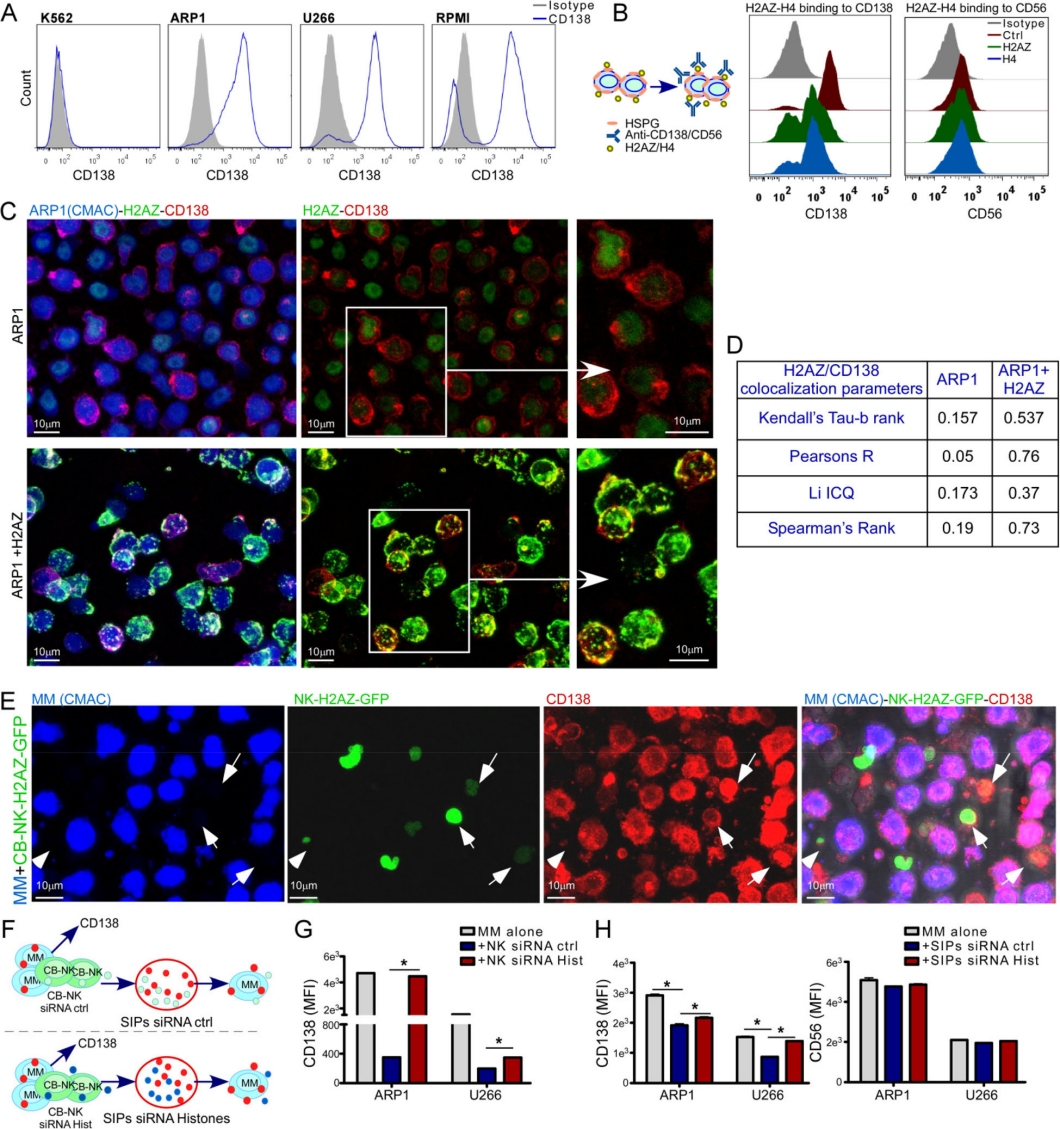
NK-histones specifically bind to CD138 on MM cell surface. **a**. CD138 expression in MM and non-MM K562 cells. **b**: MM cells incubated with either H2AZ or H4 for 1 h and stained for CD138 and CD56. **c**-**d**: confocal microscopy images (**c**) and colocalization analysis (**d**) of CD138 and H2AZ after addition of recombinant H2AZ to ARP1 MM cells. **e**. CB-NK overexpressing H2AZ-GFP co-cultured with MM cells showing co-localization of H2AZ-GFP and CD138. **f**. MM cells and CB-NK (either ctrl or with knock-down of histones H2AZ, H4 and H1.5) were co-cultured for 3 h with MM cells and CD138 expression was analyzed (**g**). **h**. SIPs from co-cultures in **f** were added into MM cells alone for 40 min to analyze CD138 and CD56 expression. *p < 0.05

We noticed that NK-histones adhered to MM cell surface (Fig. 2c) and a decreased CD138 expression in vivo in MM cells after H2AZ treatment (Fig. 4g). In this regard, histones are cationic proteins that can be neutralized with anionic proteins such as heparin [20]. Interestingly, CD138 is a highly anionic type of HSPG [21] greatly abundant in the surface of MM cells, required for MM tumor growth, vascularization, and metastasis, being essential for MM cells [22, 23]. Therefore, we hypothesized that cationic histones would bind to anionic CD138 in tumor cells. After confirming CD138 expression in MM cells (Fig. 5a), MM cells were treated with either recombinant H2AZ or H4 and stained for CD138 and CD56, two markers of MM cells. Histone treatment decreased only CD138 expression (Fig. 5b) suggesting that histones were binding to CD138. Confocal fluorescence microscopy demonstrated a high colocalization of CD138 and H2AZ (Fig. 5c and d). Moreover, co-culture of CB-NK overexpressing H2AZ-GFP with MM cells also demonstrated colocalization of CD138 and H2AZ (Fig. 5e).

In addition, MM cells were co-cultured with either CB-NK control or CB-NK where histones (H2AZ, H4 and H1.5) had been knocked-down (CB-NK siRNA Hist), and CD138 expression was analyzed. Moreover, SIPs from these co-cultures were taken and added into MM cells alone to analyze also their impact in CD138 expression (Fig. 5f). In both cases, CB-NK siRNA ctrl (Fig. 5g) and SIPs siRNA ctrl (Fig. 5h) decreased CD138 expression in MM cells, an effect which was reversed after knock-down of histones (Fig. 5g and h). Additionally, SIPs did not impact CD56 expression (Fig. 5h) further supporting the specific binding of CB-NK histones to CD138.

### NK-histones promote cell clustering formation required for NK cell anti-MM activity

**Fig. 6:**
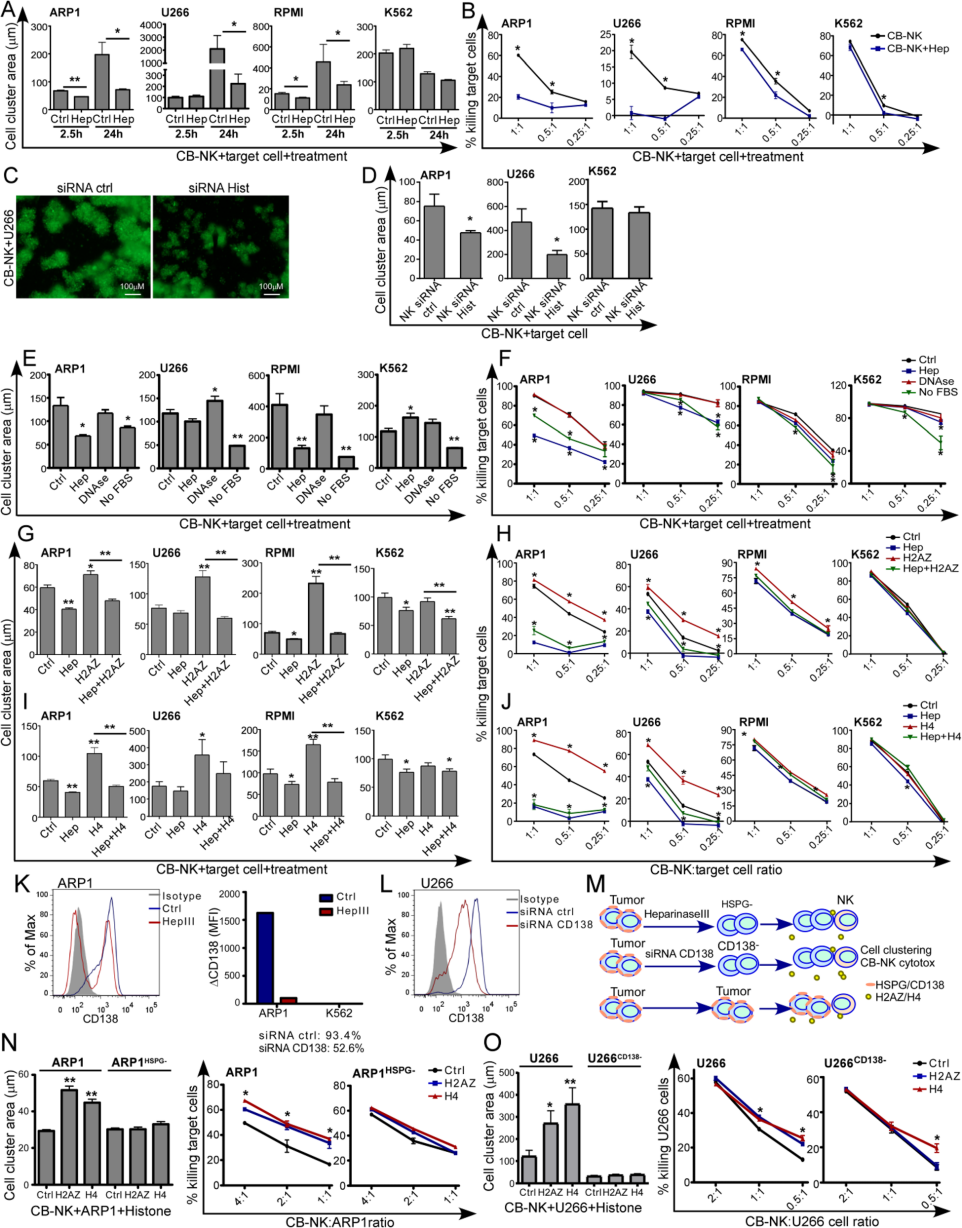
NK histones promote cell clustering formation required for NK cells anti-MM activity. **a**: Cell clustering formation between CB-NK and MM-GFP and non-MM K562-GFP cells at 2.5 h and 24 h analyzing in parallel the impact of heparin (Hep) (see also Additional file 1: Figure S6A). **b**: 24 h cytotoxicity assays values obtained from co-cultures in **a**. **c** and **d**: Cell clustering formation at 2.5 h of co-culturing MM-GFP and non-MM K562-GFP cells with either CB-NK ctrl (siRNA ctrl) or CB-NK with knock-down of histones (siRNA Hist). Bar size indicates 100 μm. **e** and **f**: Impact of DNase and absence of Fetal bovine serum (FBS) in cell culture media on cell clustering formation (**e**) and CB-NK cytotoxicity (**f**) against MM and non-MM K562 cells. Heparin was added in parallel as control. **g**-**j**: Impact of recombinant H2AZ (**g** and **h**) and H4 (**i** and **j**), in the formation of cell clustering and in CB-NK cytotoxicity against MM and non-MM K562 cells. Cell clustering analysis was measured at 2.5 h (**g** and **i**) and cytotoxicity at 24 h (**h** and **j**). See also Additional file 1: Figure S6B. **k-o**: CD138 impact on histones activity. **k-l**: CD138 expression in MM and K562 cells after Heparinase III treatment for 1-2 h (**k**) and after CD138 knock-down with siRNAs (**l**). **m**. Tumor cells ctrl, after Heparinase III treatment ^(HSPG-)^ and after CD138 knock-down (CD138^−^) were co-cultured with CB-NK adding exogenous H2AZ or H4 and cell clustering formation was evaluated at 2 h (**n**) and cytotoxicity at 6 h (**o**) (See also Additional file 1: Figure S6C). *p < 0.05. ** *p* < 0.001

Proteomic data showed a high number of released NK inflammatory proteins in the SIPs, including histones, which were also involved in coagulation (Fig. 1d). The innate immune system has the ability to initiate a process termed ‘immunothrombosis’, whereby the release of inflammatory proteins, including histones, a local intravascular scaffold is provided to immobilize, contain and destroy pathogenic microorganisms [12, 24]. Interestingly, NK cells require high effector (E):target (T) ratio for the NK and target cells to be in close proximity for optimal anti-tumor efficacy. However, NK cells represent 1–6% of peripheral blood leukocytes, therefore a process that can promote a high localized E:T ratio bringing NK and MM cells into close proximity would be physiologically relevant. Therefore, we hypothesized that histones were required for NK to promote cell clustering thereby improving MM cell killing. It is known that heparin inhibits immunothrombus formation, neutralizes cationic histones [20] and also competes with anionic HSPG for their binding to cationic ligands [11], such as histones. Therefore, 24 h cytotoxicity assays with heparin were performed at low E:T ratios, and the area of cell clusters were measured at 2.5 h and at 24 h. Heparin inhibited cell cluster formation (Additional file 1: Figure S6A and Fig. 6a) and reduced the anti-MM CB-NK activity (Fig. 6b), indicating that CB-NK requires early cluster formation to perform anti-MM activity, as it has been observed for NK antimicrobial activity [7]. For K562, the effect of heparin was minimal. The absence of HLA-I expression by K562 cells makes these cells strong targets for NK even in the absence of the improved cell-cell contact provided by cell clustering. Moreover, knock-down of histones in CB-NK also decreased cell clusters formation at 4 h of co-culturing CB-NK and MM cells at low E:T ratios, an effect not observed for K562 (Fig. 6c and d), confirming that CB-NK histones are involved in the formation of these cell clusters which are inhibited with heparin.

Furthermore, histones co-localize with DNA and were found in the SIPs with other pro-inflammatory and pro-coagulant proteins (Fig. 1d), some of which may originate from serum in the culture medium. Therefore, the effects of both DNAse treatment and removal of serum were tested. Removal of serum reduced cell clustering formation (Fig. 6e) and CB-NK cytotoxicity against MM (Fig. 6f). The serum impact was also observed for K562 (Fig. 6e and f), suggesting that the serum provides other relevant proteins in addition to CB-NK histones. DNAse treatment, on the other hand, did not impact CB-NK cytotoxicity against MM or K562 cells (Fig. 6e and f) suggesting a histone activity independent of DNA. Interestingly, DNAse treatment increased cell clustering in some cases (U266 in Fig. 6e).

In addition, same experiments were performed adding recombinant H2AZ or H4 at a non-toxic dose neither for CB-NK nor for target cells (Additional file 1: Figure S5A-C). The addition of H2AZ or H4 increased the size of cell clusters (Additional file 1: Figure S6B, Fig. 6g and i) and the CB-NK anti-MM activity (Fig. 6h and j), being this effect abrogated by the addition of heparin (Fig. 6g to j). Consistently, heparin, H2AZ and H4 impact in the CB-NK cytotoxicity against K562 was much lower or non-detectable (Fig. 6g to j).

Last, to confirm interaction of CD138 and histones in cell cluster formation and in CB-NK cytotoxicity, the effect of histones was evaluated in the absence of CD138 after Heparinase III treatment [11] or knock-down of CD138 by siRNAs. Both heparinase III and siRNA treatment reduced CD138 expression in MM cells (Fig. 6k and l). Tumor cells (ARP1 and K562) after Heparinase III treatment (ARP1^HSPG-^ and K562^HSPG-^) and MM cells after knock-down of CD138 (siRNA) were co-cultured in parallel with CB-NK, adding exogenous recombinant H2AZ and H4 at a non-toxic dose (Fig. 6m). Heparinase III treatment and CD138 knock-down abolished the impact of histones in cell cluster formation and in CB-NK anti-MM activity (Fig. 6n and o) without changes in cluster formation and cytotoxicity against K562 cells (Additional file 1: Figure S6C).

### Released NK-histones also promote T cell/MM cell clustering increasing T cell anti-MM activity

**Fig. 7:**
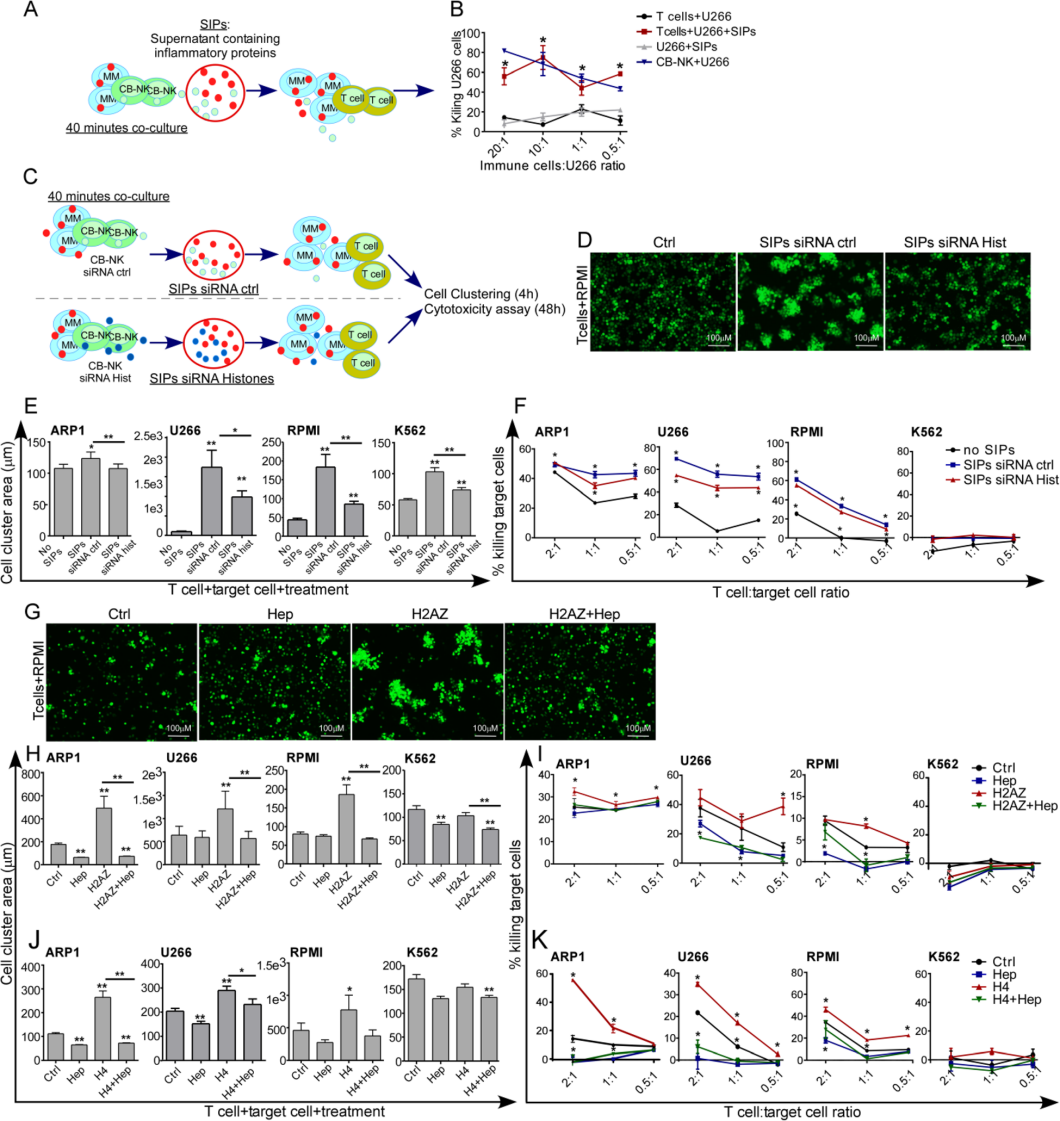
Released NK cell histones also promote T cell/MM cell clustering increasing T cell anti-MM activity: **a.** CB-NK and MM cells were co-cultured and the SIPs were collected and added into 3 h cytotoxicity assays against MM cells adding as effectors T cells (**b**). SIPs were added in parallel to U266 cells alone as control, and CB-NK were also included as effectors to compare the efficacy. **c-f**: Impact of histones in the SIPs capacity to increase T cell anti-tumor activity. CB-NK ctrl (siRNA ctrl) and CB-NK with knockdown of histones (siRNA Hist) were co-cultured with MM cells to obtain both SIPs siRNA ctrl and SIPs siRNA Hist (**c**). These SIPs were added into T-cell/tumor cell co-cultures, and the impact on cell clustering formation after 4 h (**d-e)** and on T cell cytotoxicity after 48 h was analyzed (**f**). **g**-**k**: Impact of recombinant H2AZ (**g-i**) and H4 (**j** and **k**), in the formation of cell clustering after 4 h, and in the T cell cytotoxicity vs MM and non-MM K562 cells after 48 h. Heparin was added in parallel. *p < 0.05. ** *p* < 0.001

As NK-histones were detected in the SIPs (Fig. 1d) and given that NK cells can shape the antitumor activity of T lymphocytes [25–28], we hypothesized that released NK-histones would also increase T cell/MM cell cluster formation facilitating anti-tumor T cell activity. Therefore, SIPs obtained from CB-NK/MM co-cultures (Fig. 7a) added into cytotoxicity assays of CD3 T lymphocytes against MM cells resulted in specifically increased anti-MM T cell activity (Fig. 7b).

To further investigate whether histones contained in the SIPs were responsible for the improved T-cell killing of MM, H2AZ, H4 and H1.5 were knocked down in CB-NK that were co-cultured with MM cells. SIPs obtained from these co-cultures were added into 48 h cytotoxicity assays with T cells at low E:T ratios, measuring area of cell clusters at 4 h (Fig. 7c). Whereas SIPs from NK cells with siRNA control increased the area of T cell/MM cell clusters (Fig. 7d and e) as well as T cell anti-MM activity (Fig. 7f); SIPs from NK with silenced histones showed diminished effects (Fig. 7d-f), confirming that histones released from NK cells increase also T cell-MM cell clustering and T cell anti-MM activity. For K562, even though SIPs increased cell cluster formation between T cells and K562 cells, they could not increase T cell cytotoxicity since K562 cells are not a target for T cells.

We also tested whether recombinant H2AZ and H4 could also promote clustering of T cells with MM. In both cases, the area of T cell/MM cell clusters (Fig. 7g, h and j) and the anti-MM T-cell activity were increased (Fig. 7i and k) in the presence of histones H2AZ and H4. Heparin addition reversed the enhanced clustering (Fig. 7g, h and j) and tumor cell killing (Fig. 7i and k). Of note, heparin also reduced cluster formation between T-cells and K562 cells, without changes in cytotoxicity.

### Histone impact is not a common mechanism for other tumor types

Since NK-histones did not increase the anti-tumor activity of NK and T cells against K562 non-MM cells, we aimed to assess whether this histone impact was specific against MM. Thus, cytotoxicity assays of either NK or T cells revealed a very low impact of exogenous H2AZ and H4 in the NK and T cell anti-tumor activity against lymphoid B cells (Ramos) (Additional file 1: Figure S6D and E), and no impact was detected towards Jurkat T cells (Additional file 1: Figure S6D and E) indicating a specific histone activity against MM. Interestingly, heparin reduced CB-NK and T cell cytotoxicity for both Ramos and Jurkat cells (Additional file 1: Figure S6D and E), suggesting that other proteins different to histones and related to inflammation/immunothrombosis could be involved in the anti-tumor activity against Ramos and Jurkat cells. To support this hypothesis, either Ramos or Jurkat T cells were co-cultured with CB-NK to collect SIPs (Additional file 1: Figure S6F) that were added in cytotoxicity assays with T cells and tumor cells. As previously shown for MM, SIPs, containing a high number of inflammatory proteins, increased both cell cluster formation and T cell anti-tumor activity against Ramos and Jurkat cells (Additional file 1: Figure S6G and H). Taking together, our results demonstrate a novel mechanism of histone-mediated NK cytotoxicity through binding to CD138 on MM cell surface.

## Discussion

The field of immunotherapy to treat cancer has grown over recent years [29, 30]. While some strategies show remarkable success [31, 32], others, including NK-based therapies, have still yet to realize their full therapeutic potential [1]. Access to banked CB has provided a source of NK to expand NK cells to clinically-relevant doses making NK-based cellular immunotherapies a real possibility [33–35]. The mechanisms by which NK kill tumor cells are becoming better understood. Previous evidence showing the relevance of NK-tumor cell communication events in mechanisms leading to a transmissible anti-tumor activity [9], suggested us to perform TRANS-SILAC proteomics to unravel novel transmitted cytotoxic NK molecules. Surprisingly, we identified a high number of CB-NK histones actively and early transferred to MM and participating in the killing of MM cells. NK-histones, through binding to CD138 on MM cells, promoted the formation of immune-tumor cell clusters, facilitating the immune attack of not only NK cells but also T lymphocytes.

Here, TRANS-SILAC proteomic analysis and further assays showed that histones were dynamically transferred from CB-NK through different MM cells, and also released to the extracellular milieu. Histones release is a phenomenon performed by neutrophils in Neutrophil Extracelullar Traps (NETs) which consist of fibril matrix containing histones to immobilize and eliminate microbial pathogens [36, 37]. We confirmed that NK cells also release histones in vesicles and in similar structures to NETs, and that in addition to their well-known antimicrobial activity [36, 37], H2AZ and H4 per se exerted a growth inhibitory effect in MM cells in vitro and also in vivo for H2AZ, being in agreement with previously described anti-tumor properties of histones [15, 38]. In addition to this growth inhibitory effect, we found that histones are involved in CB-NK anti-MM activity as part of the cytotoxic arsenal of NK cells to perform anti-MM activity.

The existence of an immunoregulatory NK cell subpopulation [39] suggests a relevant role for this NK pro-inflammatory activity [19]. In this regard, NK cells release pro-inflammatory granulysin and granzymes, whose role is not completely understood [40], and cytokines which coordinate the immune response by recruiting DCs [25–28] and promoting T-cell activity [41]. However, effector cytokines secreted by NK cells are detected after 2–3 h of immune cell-tumor cell contact, suggesting the existence of other molecules released earlier and responsible for initiating these processes. Here, we provide evidence of the pro-inflammatory nature of NK histones [18, 36] by activating pyroptosis [13, 18, 42] in MM. Histones in collaboration with other pro-inflammatory molecules form the first line of defense to eliminate pathogens by initiating immunothrombosis, a complex process where neutrophil histones contribute to generate an intravascular scaffold for containment, exposure and destruction of pathogens [12, 24]. This process, illustrating the relevance of multi-cellular clusters to enable cross-talk between immune cells to eliminate microbial pathogens [7, 8], appears analogous to the capacity of histones to create both NK/tumor and T-cell/tumor clusters to improve NK and T cell anti-MM activity. This additional immunoregulatory ability of NK cells should be considered in immunotherapy strategies, especially given the fact that NK cells constitute 1–6% of leukocytes in PB. Importantly, DCs promote clustering and NK cell activation after bacterial infection [7] leading to T cell activation, a process which is detected after a few hours [7]. Here, we demonstrate that histones secreted by NK cells at early time points contribute to this effect in the absence of DCs, suggesting a role for other proteins as initiators of the whole effector immune response.

In addition, our proteomic approach provided with a method to detect NK proteins with anti-MM activity, suggesting the use of this technique to detect specific anti-tumor proteins. The observed anti-MM activity of NK-histones was proposed to be mediated through binding to CD138, a HSPG present on the surface of MM cells [43]. HSPGs are composed of proteins bound to polyanionic heparan sulfate chains that provide additional negative charges to the already anionic nature of lipid membranes [44]. These negative charges enable HSPG-mediated endocytosis of macromolecules. Specifically, HSPG can uptake arginine and lysine rich peptides [45, 46], which interestingly are highly enriched in histones [47], and could explain the observed histone affinity for CD138. The disappearance of histone impact after CD138 knock-down, and after heparin treatment, a competitor of HSPG for their binding to cationic ligands [11], and the co-localization of histones with CD138 confirmed that histone activity requires binding to CD138 on MM cells. Even though we only analyzed the effect of histones over CD138, NK histones might bind to other types of HSPG and should require further investigation.

In conclusion, this study demonstrates by first time a new anti-MM mechanism of CB-NK mediated by early histone-transfer and release, where histones bind to CD138 promoting immune-tumor cell clustering facilitating both NK and T cell anti-tumor activity. Considering the poor clinical results infusing NK cells, this additional role of NK cells modulating T lymphocytes could open up new avenues for future immunotherapy studies.

## Supplementary information

**Additional file 1.** Supplementary Figures and Supplementary Methods. (DOCX 3719 kb)

## Data Availability

All data generated or analysed during this study are included in this published article and its supplementary information files.
