# Supplementary material for: Extracellular NK histones promote immune cell anti-tumor activity by inducing cell clusters through binding to CD138 receptor
Source: J Immunother Cancer. 2019 Oct 16;7:259. doi: 10.1186/s40425-019-0739-1 (PMC6794915; doi:10.1186/s40425-019-0739-1)
Supplement: Supplementary file 1 — Additional file 1. Supplementary Figures and Supplementary Methods. (DOCX 3719 kb) [file 40425_2019_739_MOESM1_ESM.docx]

**Supplementary information:**

**
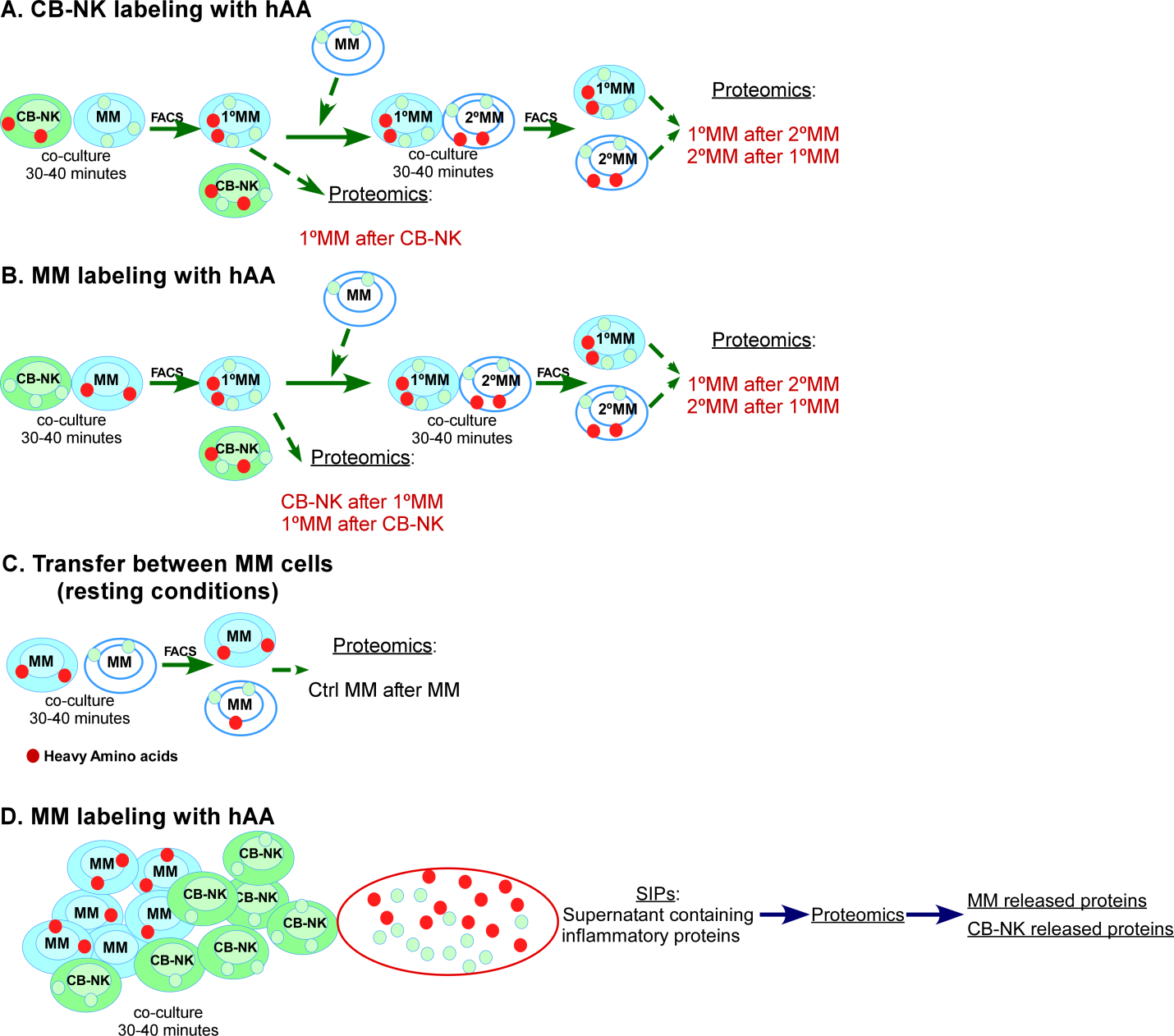
Figure S1: Labeling and sorting strategies used to obtain TRANS-SILAC proteomics results. A:** Cord-blood derived NK cells (CB-NK) were expanded with heavy amino acids (hAA) to acquire a heavy proteome. Then, 20x10^6^ of CB-NK (Bodipy-labeled, in green) and 10x10^6^ of multiple myeloma (MM) cells (CMAC-labeled, in blue) were co-cultured for 40 minutes and both populations were FACS sorted. Half of the 1ºMM cells were left alone (1º MM after CB-NK), and half of them were co-cultured for 40 minutes with fresh unstained MM cells (2º MM) at a 1:1 ratio. Then, both of them were FACS sorted, obtaining “1ºMM after 2ºMM”, and “2ºMM after 1ºMM” cell populations. 1x10^6^ of each of these cell populations were kept in cell pellet at -80ºC for further proteomic analysis. **B**: MM cells were expanded with hAA to acquire a heavy proteome. Then, 20x10^6^ of CB-NK (Bodipy-labeled in green) and 10x10^6^ of MM cells (CMAC-labeled in blue were co-cultured and FACS sorted as in **A**. The different cell populations obtained (CB-NK, 1ºMM after CB-NK, 1ºMM after 2ºMM and 2ºMM after 1ºMM) underwent proteomic analysis. **C**: **Basal levels of MM cell-cell communication**. As control for MM proteome transfer (absence of CB-NK), 10x10^6^ of hAA labeled MM cells in blue (CMAC) were co-cultured for 40 minutes with 10x10^6^ of unstained MM cells, both populations were FACS sorted and subjected to proteomic analysis. MM cells were the ARP1 cell line. **D**: 5x10^6^ of hAA labeled MM cells were co-cultured with 10x10^6^ of CB-NK for 40 minutes and the supernatant, termed Supernatant containing inflammatory proteins (SIPs), was collected to perform TRANS-SILAC proteomics and determine both CB-NK and MM released proteins.

**
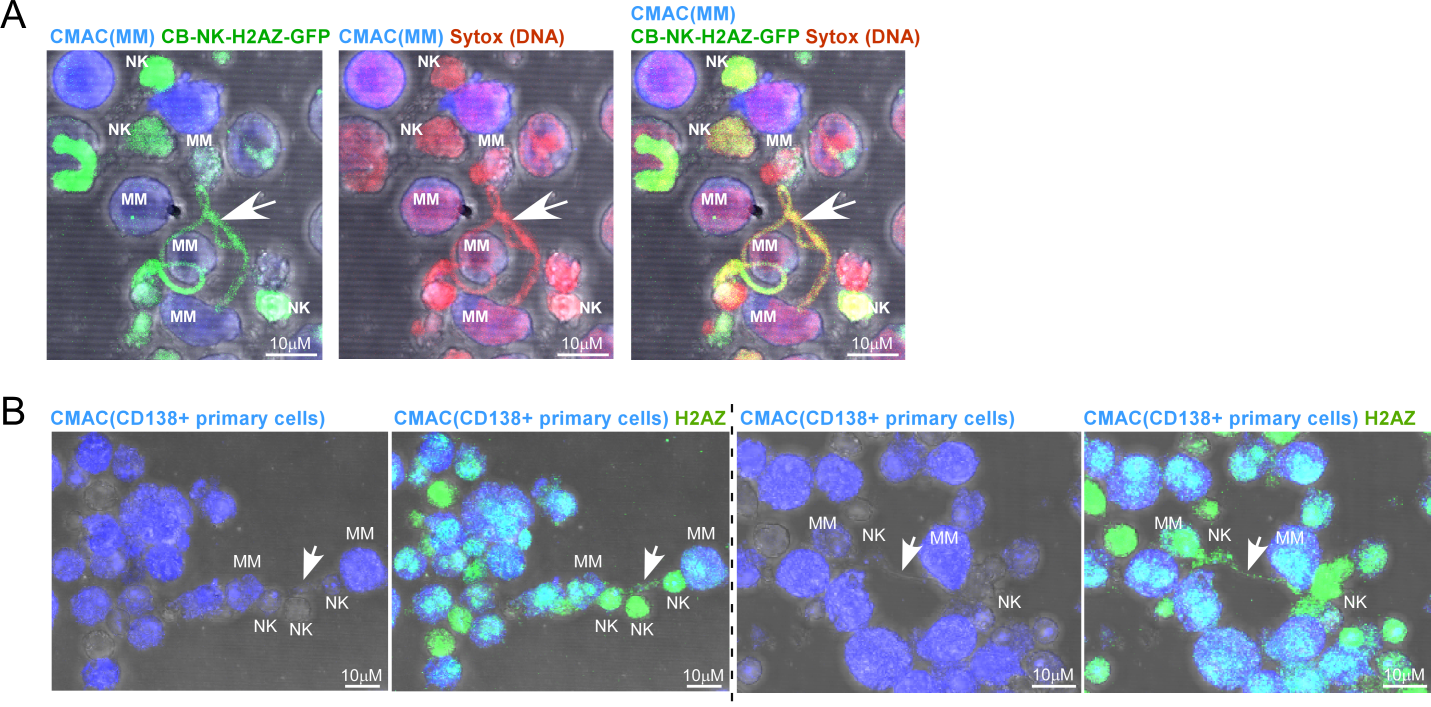
**

**Figure S2:** **A**. Co-localization of H2AZ with DNA: MM cells in blue (CMAC) with CB-NK over-expressing H2AZ-GFP (in green) and Sytox (in red) for DNA staining. Arrow indicates one H2AZ**-**intercellular structures between MM cells containing H2AZ-GFP from CB-NK with DNA. **B**. Primary MM CD138^+^ cells in blue (CMAC) co-cultured with CB-NK, adding H2AZ antibody (in green).


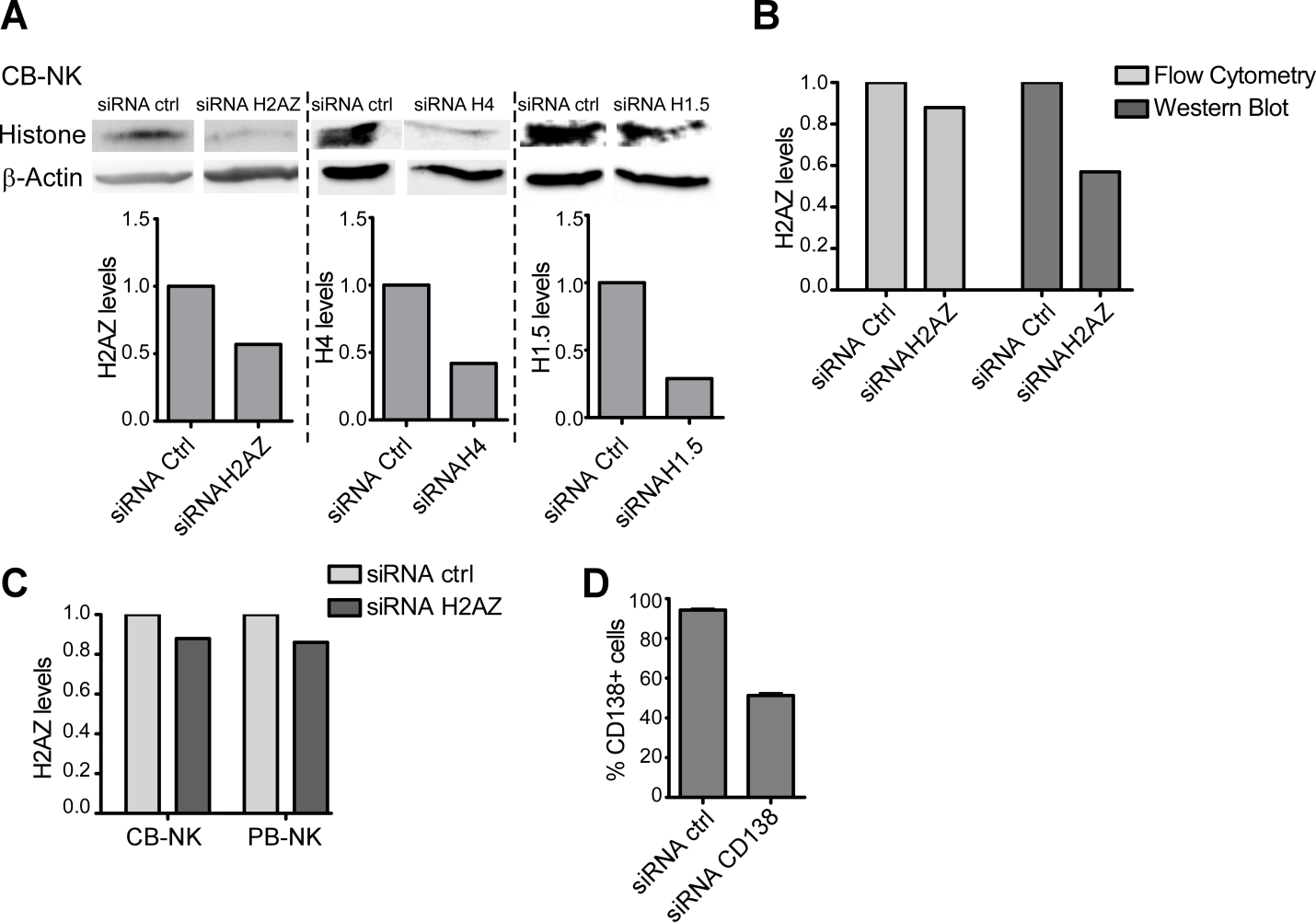


**Figure S3: siRNA transfection efficiencies. A**. Histone expression analyzed by Western Blot after silencing (siRNA) H2AZ, H4 and H1.5 in CB-NK. **B**. H2AZ expression after silencing H2AZ and analyzed in parallel by flow cytometry and WB. **C**. H2AZ expression after silencing H2AZ in CB-NK and peripheral blood-NK cells (PB-NK) analyzed by flow cytometry. **D**. Percentage of CD138+ cells after silencing CD138 in U266-MM cells analyzed by flow cytometry. Values in **A,** **B** and **C** have been normalized *vs* siRNA ctrl.

**
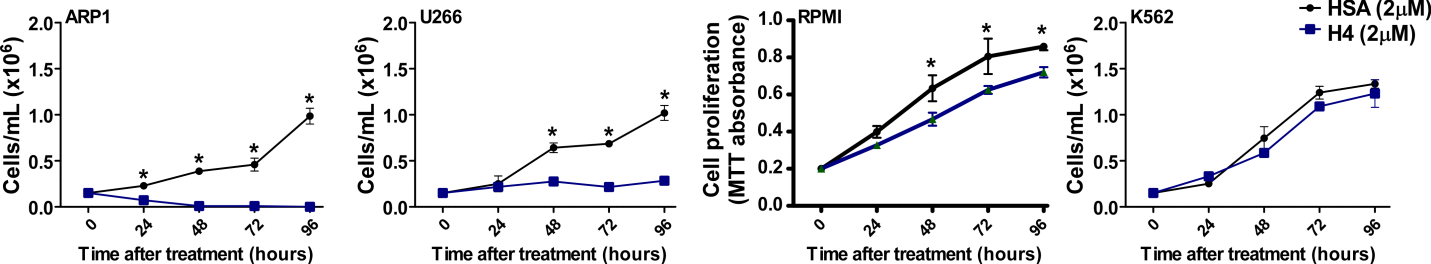
**

**Figure S4: Impact of recombinant H4** on viability of MM (ARP1, U266 and RPMI) and non-MM K562 cells. HSA: Human Serum Albumin (HSA) was added as protein control in parallel with Recombinant H4. Cell proliferation was measured by viable cell count. *p<0.05.

**
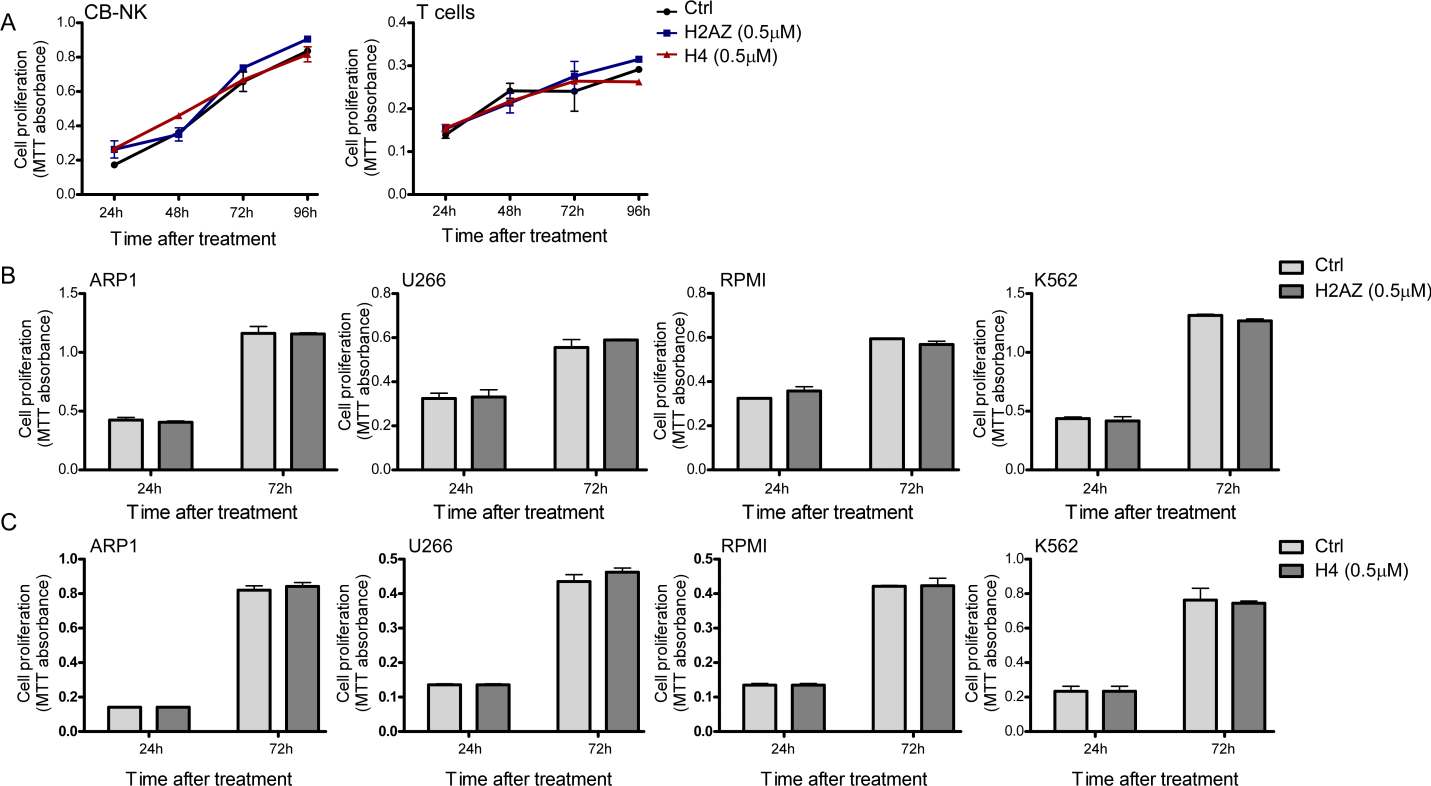
**

**Figure S5: A**. **Impact of recombinant H2AZ and H4 at a dose of 0.5 µM** on CB-NK and T cell proliferation during four days measured by viable cell count over 4 days. **B** and **C**: Impact of recombinant H2AZ (**B**) and H4 (**C**) at a dose of 0.5 µM on MM (ARP1, U266, RPMI) and non-MM K562 cells measured by MTT absorbance.

**
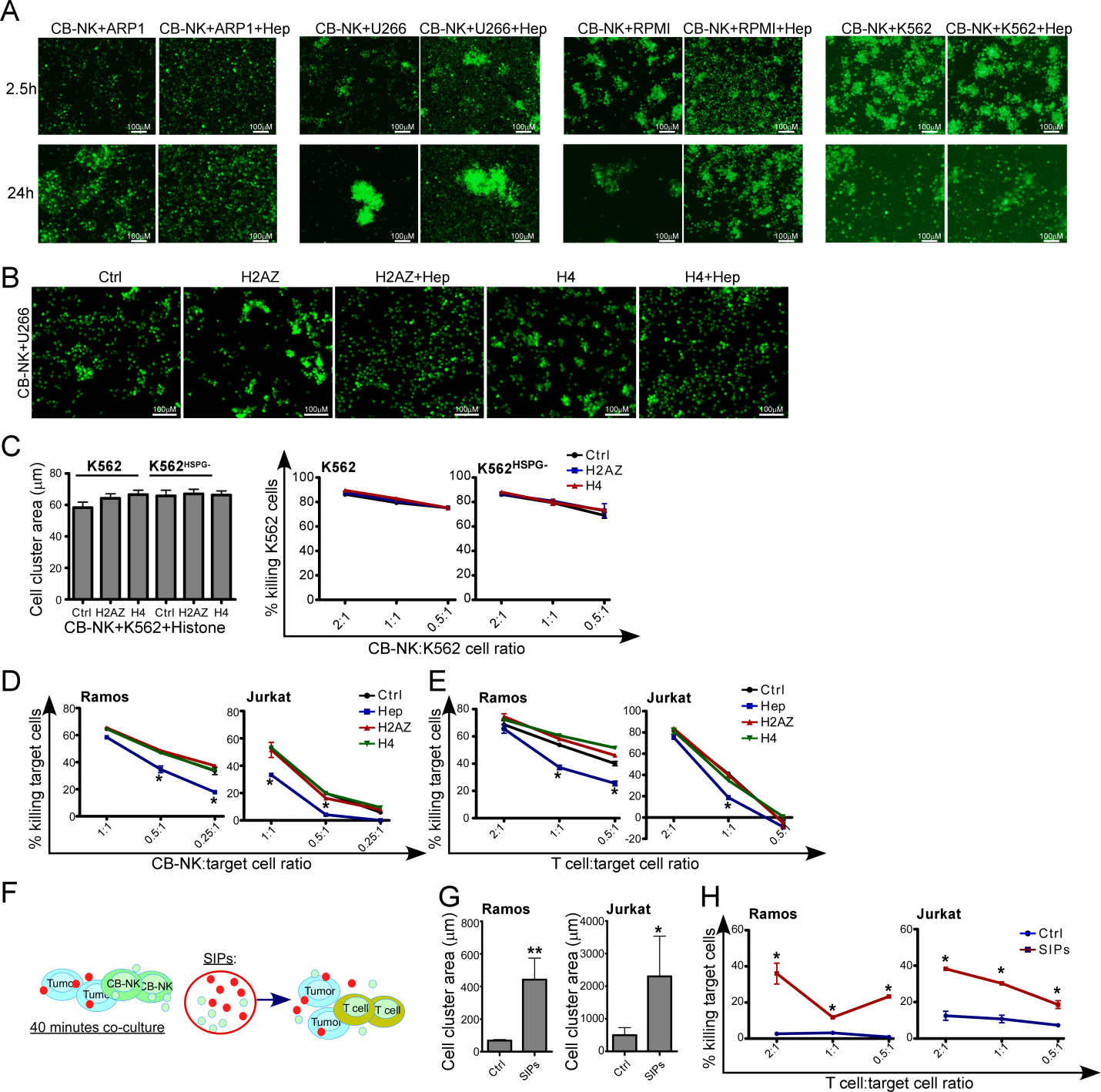
**

**Figure S6: A-B. Cell clustering formation between CB-NK and MM-GFP** (ARP1, U266, RPMI) a**nd non-MM K562-GFP** cells analyzing in parallel the impact of heparin (Hep) at 2.5h and 24h (**A**), and the impact of recombinant H2AZ and H4 at 4h with and without Hep in U266 cells (**B**). H2AZ and H4 were added at 0.5 µM. Bar size indicates 100 μm. **C**. **Impact of HSPG in cell cluster formation (2h) and CB-NK cytotoxicity (6h) against K562 cells**. K562 cells ctrl and after Heparinase III treatment ^(HSPG-)^ were co-cultured with CB-NK adding exogenous H2AZ or H4. **D-H:** **Histone impact is not a common mechanism for other tumor types**. Impact of heparin and recombinant H2AZ and H4 in CB-NK (**D**) and T cell (**E**) cytotoxicity against non-MM (Ramos and Jurkat) cells. **F**-**H**: Impact of SIPs on cell clustering formation (**G**) and on T cell cytotoxicity (**H**) of T cells against Ramos and Jurkat Cells. SIPs were obtained co-culturing CB-NK with tumor cells (**F**). Cytotoxicity assays from D, E and H were performed at 12h.

**Tables S1-S6: Transferred proteins between cells identified after applying the following filters: *1)* FDR=0.01, *2)* presence of labeled AA, and *3)* PSMs≥2**. All proteins are ordered from higher to lower PSMs.

**Table S1:** CB-NK proteins transferred to 1ºMM (1ºMM after CB-NK).

| **1ºMM after CB-NK** | | | | | |
| --- | --- | --- | --- | --- | --- |
| **Swiss-Prot number** | **Swiss-Prot name** | **Swiss-Prot number** | **Swiss-Prot name** | **Swiss-Prot number** | **Swiss-Prot name** |
| P04114 | APOB | P09874 | PARP1 | Q95604 | 1C17 |
| P60709 | ACTB | Q96KK5 | H2A1H | P20645 | MPRD |
| P04406 | G3P | P34932 | HSP74 | P83731 | RL24 |
| P08238 | HS90B | P06454 | PTMA | P16401 | H15 |
| P11142 | HSP7C | P23246 | SFPQ | Q12840 | KIF5A |
| P06733 | ENOA | P05387 | RLA2 | Q29RF7 | PDS5A |
| P62805 | H4 | P35580 | MYH10 | Q7KZ85 | SPT6H |
| P68133 | ACTS | P06899 | H2B1J | Q8TED0 | UTP15 |
| P07437 | TBB5 | Q6NXT2 | H3C | Q8IUI8 | CRLF3 |
| P07900 | HS90A | A6NNZ2 | TBB8L | P19012 | K1C15 |
| P35579 | MYH9 | Q9BUF5 | TBB6 | Q9UJY5 | GGA1 |
| P68371 | TBB4B | P0C0S5 | H2AZ | Q5T4W7 | ARTN |
| Q562R1 | ACTBL | Q15365 | PCBP1 | Q9Y6N7 | ROBO1 |
| P68104 | EF1A1 | P48643 | TCPE | O95478 | NSA2 |
| P63104 | 1433Z | Q8WU39 | MZB1 | P11717 | MPRI |
| P22626 | ROA2 | P14317 | HCLS1 | Q96NW4 | ANR27 |
| P60174 | TPIS | P06753 | TPM3 | A2VEC9 | SSPO |
| P61978 | HNRPK | P26010 | ITB7 | Q8IZF2 | GP116 |
| P31146 | COR1A | P62158 | CALM | O94854 | K0754 |
| P16403 | H12 | P54819 | KAD2 | Q5JPB2 | ZN831 |
| P23528 | COF1 | P60660 | MYL6 | Q6ZS30 | NBEL1 |
| P07737 | PROF1 | A6NHL2 | TBAL3 | Q9BZJ7 | GPR62 |
| O60814 | H2B1K | P09382 | LEG1 | Q9UKP6 | UR2R |
| P08670 | VIME | Q01081 | U2AF1 | P55291 | CAD15 |
| Q00839 | HNRPU | P13797 | PLST | Q9P2D7 | DYH1 |
| P13796 | PLSL | Q14683 | SMC1A | P12544 | GRAA |
| P23284 | PPIB | O76021 | RL1D1 |  |  |
| Q58FF6 | H90B4 | P56537 | IF6 |  |  |

**Table S2:** CB-NK proteins transferred from 1ºMM to 2ºMM cells (2ºMM after 1º MM).

| **2º MM after 1ºMM** | | | |
| --- | --- | --- | --- |
| **Swiss-Prot number** | **Swiss-Prot name** | **Swiss-Prot number** | **Swiss-Prot name** |
| P60709 | ACTB | P46063 | RECQ1 |
| P04406 | G3P | A4UGR9 | XIRP2 |
| P62805 | H4 | Q12797 | ASPH |
| P68032 | ACTC | Q15746 | MYLK |
| Q96KK5 | H2A1H | Q52LJ0 | FA98B |
| Q00839 | HNRPU | Q86XP3 | DDX42 |
| P0C0S5 | H2AZ | Q8WZ42 | TITIN |
| Q562R1 | ACTBL | O94854 | K0754 |
| Q8IUE6 | H2A2B | O75363 | BCAS1 |
| P07355 | ANXA2 | P01042 | KNG1 |
| P31146 | COR1A | P78413 | IRX4 |
| P62263 | RS14 | Q86VR7 | VS10L |
| Q96MG7 | MAGG1 | Q8N1G2 | CMTR1 |
| Q9HC10 | OTOF | Q8N319 | CF223 |
| Q9P0X4 | CAC1I | Q9UJ04 | TSYL4 |
| P33241 | LSP1 | P28799 | GRN |
| Q00688 | FKBP3 |  |  |

**Table S3:** CB-NK proteins remaining in 1ºMM cells after being co-cultured with 2ºMM cells (1º MM after 2ºMM).

| **1ºMM after 2ºMM** | | | |
| --- | --- | --- | --- |
| **Swiss-Prot number** | **Swiss-Prot name** | **Swiss-Prot number** | **Swiss-Prot name** |
| P27348 | 1433T | Q03001 | DYST |
| P68104 | EF1A1 | Q9Y263 | PLAP |
| P04350 | TBB4A | Q9Y2D5 | AKAP2 |
| P07900 | HS90A | P07108 | ACBP |
| P15531 | NDKA | P54105 | ICLN |
| P09874 | PARP1 | O75683 | SURF6 |
| Q00839 | HNRPU | P30455 | 1A36 |
| A6NNZ2 | TBB8L | Q9BRX2 | PELO |
| P60842 | IF4A1 | P43246 | MSH2 |
| P52272 | HNRPM | Q9NYJ7 | DLL3 |
| P35580 | MYH10 | O60524 | NEMF |
| O43390 | HNRPR | Q5C9Z4 | NOM1 |
| Q8WU39 | MZB1 | Q8WVM8 | SCFD1 |
| Q14240 | IF4A2 | Q96EK7 | F120B |
| Q06830 | PRDX1 | O15021 | MAST4 |
| Q86VM9 | ZCH18 | Q2M2I5 | K1C24 |
| Q9HB71 | CYBP | Q8N130 | NPT2C |
| P13797 | PLST | Q8N3J5 | PPM1K |
| O60610 | DIAP1 | Q8NFT6 | DBF4B |
| Q9H6Z4 | RANB3 | Q9BW71 | HIRP3 |

**Table S4:** MM proteins transferred to CB-NK (CB-NK after 1ºMM).

| **CB-NK after 1ºMM** | | | |
| --- | --- | --- | --- |
| **Swiss-Prot number** | **Swiss-Prot name** | **Swiss-Prot number** | **Swiss-Prot name** |
| P60709 | ACTB | P22626 | ROA2 |
| P62805 | H4 | P07900 | HS90A |
| P07437 | TBB5 | Q562R1 | ACTBL |
| Q16777 | H2A2C | P68431 | H31 |
| P04908 | H2A1B | P84243 | H33 |
| P58876 | H2B1D | P09651 | ROA1 |
| P68032 | ACTC | P27348 | 1433T |
| P04406 | G3P | P60174 | TPIS |
| P23527 | H2B1O | Q00839 | HNRPU |
| Q6S8J3 | POTEE | P06748 | NPM |
| P68363 | TBA1B | P14625 | ENPL |
| P16104 | H2AX | Q14103 | HNRPD |
| P68104 | EF1A1 | P23284 | PPIB |
| P04350 | TBB4A | P07910 | HNRPC |
| P0C0S5 | H2AZ | P40926 | MDHM |
| P63104 | 1433Z | P07237 | PDIA1 |
| P06576 | ATPB | Q15084 | PDIA6 |
| P11021 | GRP78 | P61604 | CH10 |
| P25705 | ATPA | P26373 | RL13 |
| P10809 | CH60 | P30086 | PEBP1 |
| P07737 | PROF1 | P05538 | DQB2 |
| P08238 | HS90B | Q86U86 | PB1 |
| Q71DI3 | H32 |  |  |

**Table S5:** MM proteins transferred from 1ºMM to 2ºMM in resting conditions **(ctrl MM after MM).**

| **Ctrl MM after MM** | |
| --- | --- |
| **Swiss-Prot number** | **Swiss-Prot name** |
| O60814 | H2B1K |
| P04406 | G3P |
| P62805 | H4 |
| P17066 | HSP76 |
| Q99832 | TCPH |
| Q58FG1 | HS904 |

**Table S6:** MM proteins transferred from 1ºMM (after CB-NK exposure) to 2ºMM.

| **1ºMM (after CB-NK) to 2ºMM** | | | | | | | |
| --- | --- | --- | --- | --- | --- | --- | --- |
| **Swiss-Prot number** | **Swiss-Prot name** | **Swiss-Prot number** | **Swiss-Prot name** | **Swiss-Prot number** | **Swiss-Prot name** | **Swiss-Prot number** | **Swiss-Prot name** |
| P60709 | ACTB | Q58FG1 | HS904 | P50570 | DYN2 | P27482 | CALL3 |
| P04406 | G3P | P52597 | HNRPF | Q96KC8 | DNJC1 | P48723 | HSP13 |
| P68104 | EF1A1 | P63104 | 1433Z | Q86V81 | THOC4 | Q12926 | ELAV2 |
| P10809 | CH60 | Q00839 | HNRPU | Q12888 | TP53B | Q6ZRI0 | OTOG |
| P08238 | HS90B | P54652 | HSP72 | Q9UHD9 | UBQL2 | Q9HC10 | OTOF |
| P14618 | KPYM | P26599 | [PTBP1 | Q5TFE4 | NT5D1 | Q15154 | PCM1 |
| P04075 | ALDOA | P43243 | MATR3 | Q15397 | K0020 | Q96EK5 | KBP |
| P68133 | ACTS | P52272 | HNRPM | Q6Y7W6 | PERQ2 | Q8WZ42 | TITIN |
| P68032 | ACTC | Q13263 | TIF1B | P43034 | LIS1 | A6NFX1 | MFS2B |
| P07437 | TBB5 | P12814 | ACTN1 | Q9GZZ1 | NAA50 | P25774 | CATS |
| P07900 | HS90A | P35580 | MYH10 | Q63HN8 | RN213 | Q02223 | TNR17 |
| P11142 | HSP7C | A6NMY6 | AXA2L | Q01130 | SRSF2 | Q8N319 | CF223 |
| Q9BQE3 | TBA1C | P46940 | IQGA1 | P30876 | RPB2 | Q92800 | EZH1 |
| P06576 | ATPB | Q8WU39 | MZB1 | P35908 | K22E | O43865 | SAHH2 |
| P62805 | H4 | P06744 | G6PI | Q13242 | SRSF9 | O60449 | LY75 |
| Q71U36 | TBA1A | A6NKZ8 | YI016 | Q9BY11 | PACN1 | P12111 | CO6A3 |
| Q16777 | H2A2C | P49368 | TCPG | Q8N3U4 | STAG2 | P30825 | CTR1 |
| P68371 | TBB4B | P48643 | TCPE | Q9NR31 | SAR1A | P49840 | GSK3A |
| P04908 | H2A1B | P30086 | PEBP1 | P09601 | HMOX1 | P57052 | RBM11 |
| P04350 | TBB4A | Q15233 | NONO | Q96SY0 | VWA9 | Q15413 | RYR3 |
| Q13885 | TBB2A | Q13310 | PABP4 | Q4V328 | GRAP1 | Q2VIQ3 | KIF4B |
| P22626 | ROA2 | P30040 | ERP29 | Q86YV0 | RASL3 | Q7Z589 | EMSY |
| O60814 | H2B1K | Q15149 | PLEC | Q8WX92 | NELFB | Q8NFY9 | KBTB8 |
| Q562R1 | ACTBL | P62081 | RS7 | P40222 | TXLNA | Q8WWI1 | LMO7 |
| Q15084 | PDIA6 | P31040 | DHSA | Q969Y2 | GTPB3 | Q8WXG9 | GPR98 |
| P07195 | LDHB | P55884 | EIF3B | P18583 | SON | Q969Q6 | P2R3C |
| P06899 | H2B1J | P18206 | VINC | P18077 | RL35A | Q96QI5 | HS3S6 |
| P06748 | NPM | O14980 | XPO1 | Q9H583 | HEAT1 | Q9NR48 | ASH1L |
| P16104 | H2AX | Q8N0Y7 | PGAM4 | Q9UNF0 | PACN2 | Q9Y2I7 | FYV1 |
| P09651 | ROA1 | P51991 | ROA3 | Q96BP3 | PPWD1 | O94854 | K0754 |
| P00338 | LDHA | P00367 | DHE3 | Q9Y619 | ORNT1 | P08311 | CATG |
| Q96QV6 | H2A1A | Q9NY33 | DPP3 | O95983 | MBD3 | P57060 | RWD2B |
| P27348 | 1433T | P14174 | MIF | Q6F5E8 | LR16C | Q5EG05 | CAR16 |
| Q8IUE6 | H2A2B | O15523 | DDX3Y | Q9P2R7 | SUCB1 | Q5T4D3 | TMTC4 |
| P49327 | FAS | P62191 | PRS4 | P48382 | RFX5 | Q68DN1 | CB016 |
| P62258 | 1433E | P26368 | U2AF2 | O95059 | RPP14 | Q6P3R8 | NEK5 |
| P27797 | CALR | P53675 | CLH2 | Q9Y3I1 | FBX7 | Q96N77 | ZN641 |
| P61978 | HNRPK | P17066 | HSP76 | Q96PN6 | ADCYA | Q9BQC6 | RT63 |
| Q9Y490 | TLN1 | O14497 | ARI1A | Q6UW02 | CP20A | Q9ULE3 | DEN2A |
| P61247 | RS3A | O95202 | LETM1 | Q8NF91 | SYNE1 | Q9UM73 | ALK |
| O43707 | ACTN4 | Q9UPN3 | MACF1 | P38432 | COIL | Q9UPX6 | K1024 |
| P09874 | PARP1 | O75367 | H2AY | Q14344 | GNA13 | Q9Y2S2 | CRYL1 |
| P31146 | COR1A | P08195 | 4F2 | Q14CX7 | NAA25 | Q9Y6W3 | CAN7 |
| P00558 | PGK1 | Q7Z406 | MYH14 | Q96HW7 | INT4 |  |  |
| P0C0S5 | H2AZ | P52434 | RPAB3 | Q9H3S7 | PTN23 |  |  |

**Tables S7-S8: CB-NK and MM proteins released to the extracellular milieu after co-culture with MM cells identified after applying the following filters: *1)* FDR=0.01, *2)* presence of labeled AA, and *3)* PSMs≥2**. All proteins are ordered from higher to lower PSMs.

**Table S7:** CB-NK proteins released to the extracellular milieu after co-culture with MM cells.

| **CB-NK proteins released to the extracellular milieu** | | | | | | | |
| --- | --- | --- | --- | --- | --- | --- | --- |
| **Swiss-Prot number** | **Swiss-Prot name** | **Swiss-Prot number** | **Swiss-Prot name** | **Swiss-Prot number** | **Swiss-Prot name** | **Swiss-Prot number** | **Swiss-Prot name** |
| P60712 | ACTB | P21333 | FLNA | P16401 | H15 | Q3ZCL8 | SH3L3 |
| P06733 | ENOA | P29401 | TKT | P12814 | ACTN1 | Q3SYV4 | CAP1 |
| P09104 | ENOG | P13645 | K1C10 | P50395 | GDIB | P14317 | HCLS1 |
| P04406 | G3P | P08670 | VIME | Q5E9J1 | HNRPF | P60661 | MYL6 |
| P13639 | EF2 | P26641 | EF1G | P23284 | PPIB | Q3MHR5 | SRSF2 |
| Q3ZC07 | ACTC | P06576 | ATPB | P09382 | LEG1 | P39687 | AN32A |
| P14618 | KPYM | P27797 | CALR | Q3SZI4 | 1433T | P35579 | MYH9 |
| P11142 | HSP7C | P10103 | HMGB1 | P09972 | ALDOC | P09622 | DLDH |
| Q3SYU2 | EF2 | P50990 | TCPQ | Q32L48 | H2B1N | P33241 | LSP1 |
| P07900 | HS90A | P12004 | PCNA | P84227 | H32 | P15259 | PGAM2 |
| P08238 | HS90B | P40926 | MDHM | P67936 | TPM4 | P01857 | IGHG1 |
| P13796 | PLSL | P62803 | H4 | P00441 | SODC | P22749 | GNLY |
| P68103 | EF1A1 | P35908 | K22E | P07237 | PDIA1 | A5D785 | XPO2 |
| Q2KJD0 | TBB5 | P62261 | 1433E | Q00839 | HNRPU | Q56K14 | RLA1 |
| P10096 | G3P | Q01518 | CAP1 | Q8NC51 | PAIRB | P34897 | GLYM |
| Q3MHM5 | TBB4B | Q96KK5 | H2A1H | P08865 | RSSA | P12763 | FETUA |
| P08133 | ANXA6 | P07355 | ANXA2 | P09867 | ROA1 | O15144 | ARPC2 |
| P04075 | ALDOA | P54652 | HSP72 | Q3T054 | RAN | P30048 | PRDX3 |
| P10809 | CH60 | O60814 | H2B1K | Q01469 | FABP5 | P61284 | RL12 |
| P00558 | PGK1 | P06744 | G6PI | P46777 | RL5 | P14174 | MIF |
| P31081 | CH60 | Q9UQ80 | PA2G4 | P0C0S4 | H2AZ | Q3T0C7 | STMN1 |
| P26038 | MOES | P22314 | UBA1 | P52565 | GDIR1 | P67808 | YBOX1 |
| P07737 | PROF1 | P09211 | GSTP1 | P18669 | PGAM1 | P04083 | ANXA1 |
| P68432 | H31 | P14625 | ENPL | P01834 | IGKC | P28070 | PSB4 |
| P00338 | LDHA | P37802 | TAGL2 | Q3ZCK9 | PSA4 | P13646 | K1C13 |
| P63103 | 1433Z | P52566 | GDIR2 | P32119 | PRDX2 | Q0VCY7 | SRSF1 |
| P81947 | TBA1B | P17066 | HSP76 | P61157 | ARP3 | Q3B7M5 | LASP1 |
| P60174 | TPIS | A6NNZ2 | TBB8L | P61769 | B2MG | Q3T108 | PSB4_ |
| P62937 | PPIA | Q2HJ60 | ROA2 | P68509 | 1433F | P12544 | GRAA |
| P31146 | COR1A | P61978 | HNRPK | Q5KR47 | TPM3 | Q148J6 | ARPC4 |
| Q3T0P6 | PGK1 | P02253 | H12 | P62157 | CALM | A4FUA8 | CAZA1 |
| Q6EWQ7 | IF5A1 | P31948 | STIP1 | P08758 | ANXA5 |  |  |
| Q9BQE3 | TBA1C | P30101 | PDIA3 | O00299 | CLIC1 |  |  |
| P23528 | COF1 | Q14103 | HNRPD | Q01105 | SET |  |  |
| P10599 | THIO | P15531 | NDKA | Q92688 | AN32B |  |  |
| P08107 | HSP71 | O43707 | ACTN4 | O75083 | WDR1 |  |  |
| P02768 | ALBU | P23526 | SAHH | P35527 | K1C9 |  |  |
| P07195 | LDHB | P19338 | NUCL | P29692 | EF1D |  |  |
| Q06830 | PRDX1 | P62992 | RS27A | Q07021 | C1QBP |  |  |
| P11021 | GRP78 | P40925 | MDHC | Q13185 | CBX3 |  |  |
| P06748 | NPM | P52209 | 6PGD | P13693 | TCTP |  |  |
| P22392 | NDKB | P61981 | 1433G | P61603 | CH10 |  |  |
| P55072 | TERA | P40673 | HMGB2 | P35268 | RL22 |  |  |
| P31946 | 1433B | P40227 | TCPZ | P43490 | NAMPT |  |  |
| P04264 | K2C1 | P31943 | HNRH1 | Q15084 | PDIA6 |  |  |

**Table S8:** MM proteins released to the extracellular milieu after co-culture with CB-NK.

| **CB-NK proteins released to the extracellular milieu** | | | | | | | |
| --- | --- | --- | --- | --- | --- | --- | --- |
| **Swiss-Prot number** | **Swiss-Prot name** | **Swiss-Prot number** | **Swiss-Prot name** | **Swiss-Prot number** | **Swiss-Prot name** | **Swiss-Prot number** | **Swiss-Prot name** |
| P49327 | FAS | Q2HJ33 | OLA1 | Q3ZBH8 | RS20 | Q8WU39 | MZB1 |
| P48643 | TCPE | P30613 | KPYR | A7MB62 | ARP2 | P36873 | PP1G |
| P78371 | TCPB | P62701 | RS4X | Q3T0S6 | RL8 | P41091 | IF2G |
| P23381 | SYWC | P00491 | PNPH | P61955 | SUMO2 | P11908 | PRPS2 |
| P63243 | GBLP | P49951 | CLH1 | P05388 | RLA0 | P16070 | CD44 |
| P50991 | TCPD | P36578 | RL4 | O43776 | SYNC | P20618 | PSB1 |
| Q99832 | TCPH | P49736 | MCM2 | P25786 | PSA1 | P26373 | RL13 |
| Q01581 | HMCS1 | Q92945 | FUBP2 | P30041 | PRDX6 | P32969 | RL9 |
| Q2NKZ1 | TCPH | O14818 | PSA7 | P51858 | HDGF | P61585 | RHOA |
| P41250 | SYG | O14979 | HNRDL | Q15366 | PCBP2 | Q3T0Q6 | CNBP |
| Q9Y617 | SERC | Q3MHP1 | UB2L3 | Q56JU9 | RS24 | Q16543 | CDC37 |
| P08567 | PLEK | Q99733 | NP1L4 | P18203 | FKB1A | Q3T0I4 | THOC4 |
| P11586 | C1TC | Q13310 | PABP4 | P23196 | APEX1 | O15143 | ARC1B |
| P38646 | GRP75 | Q13838 | DX39B | Q2TBU9 | RUVB2 | Q3T035 | ARPC3 |
| Q3SZ54 | IF4A1 | Q3T0U2 | RL14 | Q5E9T9 | RTCB | O95218 | ZRAB2 |
| P61286 | PABP1 | P31153 | METK2 | P28838 | AMPL | P12956 | XRCC6 |
| P53396 | ACLY | P54577 | SYYC | Q3T0V4 | RS11 | P16989 | YBOX3 |
| Q13765 | NACA | Q3T0R1 | RS18 | Q3T0D5 | RL30 | Q3T0Y5 | PSA2 |
| P49368 | TCPG | Q862I1 | RL24 | Q15637 | SF01 | P30040 | ERP29 |
| Q99497 | PARK7 | Q5E9A3 | PCBP1 | Q99873 | ANM1 | P30566 | PUR8 |
| P33316 | DUT | Q29RK4 | RD23B | P38919 | IF4A3 | Q32PD5 | RS19 |
| P49588 | SYAC | O95433 | AHSA1 | P60228 | EIF3E | P50453 | SPB9 |
| Q16555 | DPYL2 | Q99436 | PSB7 | O95626 | AN32D | P61356 | RL27 |
| Q06323 | PSME1 | Q9Y490 | TLN1 | P14866 | HNRPL | Q3MIC0 | RL37A |
| Q3MHL7 | TCPZ | P00505 | AATM | P28072 | PSB6 | P63279 | UBC9 |
| Q9BWD1 | THIC | P11766 | ADHX | Q3T0F4 | RS10 | Q13907 | IDI1 |
| P05455 | LA | P14324 | FPPS | Q56JX6 | RS28 | Q16719 | KYNU |
| Q15181 | IPYR | P14868 | SYDC | Q3T087 | RL11 | Q96KP4 | CNDP2 |
| O18789 | RS2 | Q5E938 | EIF1 | Q13867 | BLMH | Q99729 | ROAA |
| Q3ZBZ8 | STIP1 | P22234 | PUR6 | Q86W56 | PARG | P40429 | RL13A |
| P37837 | TALDO | Q5E988 | RS5 | P01911 | 2B1F | P42766 | RL35 |
| P17174 | AATC | P53999 | TCP4 | P84080 | ARF1 | Q07020 | RL18 |
| P17987 | TCPA | P99999 | CYC | P14678 | RSMB | Q3SZ43 | UB2V2 |
| Q02878 | RL6 | P27635 | RL10 | Q12906 | ILF3 | Q9HB71 | CYBP |
| P33991 | MCM4 | P55036 | PSMD4 | O14737 | PDCD5 | Q29RH4 | THOC3 |
| P10768 | ESTD | Q28009 | FUS | P25705 | ATPA | Q3MHL8 | SMAP |
| P16152 | CBR1 | P62263 | RS14 | P30153 | 2AAA | Q3T0V7 | EDF1 |
| P31939 | PUR9 | Q3T0W9 | RL19 | P47756 | CAPZB | O75131 | CPNE3 |
| Q9P258 | RCC2 | Q5EAD2 | SERA | P06734 | FCER2 | O75326 | SEM7A |
| Q56JV9 | RS3A | Q1JQB2 | BUB3 | Q9UJU6 | DBNL | P12081 | SYHC |
| A6H767 | NP1L1 | P49591 | SYSC | Q16576 | RBBP7 | P17096 | HMGA1 |
| Q32L40 | TCPA | Q14566 | MCM6 | P05198 | IF2A | P20290 | BTF3 |
| P34932 | HSP74 | O15067 | PUR4 | P30086 | PEBP1 | P23588 | IF4B |
| P62424 | RL7A | P30085 | KCY | P49721 | PSB2 | P24666 | PPAC |
| P24534 | EF1B | P43487 | RANG | P56537 | IF6 | P48595 | SPB10 |
| Q3ZBF7 | TEBP | P49189 | AL9A1 | P61257 | RL26 | Q3T0F7 | MTPN |
| Q9H361 | PABP3 | Q3T171 | RL36 | Q9Y266 | NUDC | Q5E9D5 | DEST |
| P39023 | RL3 | Q5E9G3 | PSME2 | Q5E959 | STRAP | Q0P5K3 | UBE2N |
| A6H769 | RS7 | P13010 | XRCC5 | O95757 | HS74L | P61457 | PHS |
| Q76I81 | RS12 | P22102 | PUR2 | P00390 | GSHR | Q3T199 | RS23 |
| P49773 | HINT1 | P78417 | GSTO1 | Q3T169 | RS3 | P62318 | SMD3 |
| Q14974 | IMB1 | Q7L1Q6 | BZW1 | P32929 | CGL | Q32PB8 | RS21 |
| O75347 | TBCA | Q96AE4 | FUBP1 | P46778 | RL21 | Q3SZC0 | ERH |
| P18124 | RL7 | P00492 | HPRT | P49207 | RL34 | Q3SZR8 | SRSF3 |
| Q5E987 | PSA5 | P17844 | DDX5 | Q5EAD6 | RL15 | Q15019 | SEPT2 |
| O43175 | SERA | P25788 | PSA3 | Q15691 | MARE1 | Q16821 | PPR3A |
| O60506 | HNRPQ | A4FUD9 | MCM3 | A7MBJ5 | CAND1 | Q6PKG0 | LARP1 |
| P12268 | IMDH2 | P51991 | ROA3 | Q92804 | RBP56 | Q6ZUX3 | F179A |
| Q13263 | TIF1B | P54578 | UBP14 | Q3T051 | RL39 | Q8TB37 | NUBPL |
| Q2YDE4 | PSA6 | Q1JPH6 | IF4H | Q3SZ59 | RL36A | Q9HC38 | GLOD4 |
| Q5E958 | RS8 | O75828 | CBR3 | A5PK63 | RS17 | Q9NX55 | HYPK |
| Q04760 | LGUL | Q04446 | GLGB | P01563 | IFNA2 | Q9UI30 | TR112 |
| Q3SZD7 | CBR1 | P09525 | ANXA4 | P08243 | ASNS | Q9UKK9 | NUDT5 |
| Q92598 | HS105 | P13667 | PDIA4 | Q2KJ93 | CDC42 | Q9Y234 | LIPT |
| P50502 | F10A1 | P27816 | MAP4 | A4FUI2 | RUXE | A7MB10 | RRP5 |
| Q24JY1 | RL23A | P62753 | RS6 | Q3T057 | RL23 | Q2NKZ4 | TKTL2 |
| P07108 | ACBP | Q7KZF4 | SND1 | P62866 | RS30 |  |  |
| P26639 | SYTC | P49321 | NASP | Q3T003 | RL18A |  |  |

**Supplementary Methods**

**Liquid Chromatography-Tandem Mass Spectrometry (LC-MS/MS)**: Sorted cell pellets of the different experiments (Supplementary Figure 1) were lysed in Triethylammonium bicarbonate buffer (TEAB). Liquid samples were reduced, alquilated and digested overnight using Trypsin (Promega). The ratio enzyme protein used was 1-20. Digested samples were desalted on a C18 spin column (Thermo Scientific). Previous to the analysis samples were off line fractionated on a Bio SCX series II (Agilent). We used salt steps gradient varying from 10mM to 500mM ammonium acetate. Collected fractions were analyzed on a liquid system (nanoULTRA Eksigent), nanoC18 column using a linear gradient (0%B-30%B). Mobile phase B (97%ACN-3%H2O-0.1%Formic acid), mobile phase A (97%H2O-3%ACN-0.1%Formic acid) and a mass spectrometer (LTQ VELOS orbitrap, Thermo Scientific). **Protein Identification and Quantification**: Data were processed using Proteome Discoverer 1.4.1.14 (Thermo Fisher Scientific). For database searching, raw mass spectrometry files were submitted to the in-house HUMAN_TRYPSIN_PIG_UP_SP_r2014-02 fasta using SEQUEST version 28.0 (Thermo Fisher Scientific). The Percolator search node, a machine-learning supplement to the SEQUEST search algorithm that increases the sensitivity and specificity of peptide identifications, was also used. The criteria used to accept identification included a minimum of 2 peptides matched per protein, with a false discovery rate (FDR) of 1%. By selecting the proteins that contained hAA we identified heavy proteins transferred between cells; then, a filter criterion was applied to choose proteins that presented unique peptides with at least 2 different spectra (PSMs) and 2 peptides. Final list of proteins was submitted to Bioinformatic analysis with STRING software to identify biological processes, pathways and functional interactions.

**Confocal fluorescence microscopy and flow cytometry:** tumor cells were stained in green (Bodipy) or blue with Bodipy or CMAC cell trackers (Life Technologies). After washing, cells were co-cultured at 37ºC for different time periods, depending on the experiment. After co-culture cells were collected and analyzed for either confocal fluorescence microscopy or flow cytometry. For confocal microscopy, cells were transferred onto slides, fixed and permeabilized as previously described ([1](#_ENREF_1)). For DNA staining, Sytox Red Dead Cell Stain (Life Technologies) was added to fix cells. Primary antibodies were incubated overnight at 4ºC and secondary antibodies for 20 min at 37 ºC. Images were acquired using a Leica SP5 microscope, doing Z-stack acquisition images and applying the corresponding filters. Image analysis was performed with ImageJ software (Fiji).

**Green Fluorescent Protein (GFP)-fused protein generation:** Gateway-based cloning systems were used to insert and fuse genes of insert upstream of GFP. The procedure entailed two-step recombination reactions where, first, an Entry plasmid encoding the gene of interest was produced by recombining an attB1/attB2-flanked PCR fragment containing the gene of interest with the Donor plasmid containing attP1/attP2 recombination sites. This reaction was mediated by BP clonase. The resulting Entry clone, encoding the gene of interest flanked by attL1/attL2 sites, was recombined with Destination vectors to produce the final lentiviral Expression vectors (pLV430G). The recombination site in the destination vectors was positioned upstream of GFP to produce fusion proteins with C-terminal GFP.

**Lentivirus production for cell infection:** human 293T cells were transfected with the pLenti plasmid DNA previously produced and the virapower Packaging mix (Invitrogen). The day after, transfection media was removed and new fresh media added. Cells were left producing virus for 72 hours and afterwards the supernatant centrifuged and filtered. Viruses were stored at -80ºC until infection of cells was made. For cell infection: plates were coated with retronectin (Clontech, Takara), the day after, virus supernatant was added for 20 min at 37ºC, then cells and virus supernatant were added. The following day, fresh media was added. After 48 hours positive GFP-protein cells were sorted when needed. Efficiencies of CB-NK-H2AZ+ varied from 15 to 30 %. H2AZ increased expression after viral infection was measured by flow cytometry with H2AZ antibody (Cell Signaling Technology, Boston, MA) and secondary antibody Anti-Rabbit IgG-Alexa Fluor-647.

**siRNA transfection**: CB-NK were transfected with three different siRNAs for H2AZ (ID: 215089, 144896 and 144897), for H4 (ID: 44963, 256496 and 13275) and for H1.5 (ID: 145222, 16200 and 16118). As control, a Silencer Negative Control (Life Technologies) was used. siRNAs were added at 30nM final concentration, and Jet Pei™ HTS DNA Transfection Reagent (VWR) was used as transfection agent. Transfection was performed on days 13 and 14 of CB-NK *in vitro* expansion; then CB-NK were used on day 15 for cytotoxicity experiments. Freshly isolated Peripheral blood NK cells (PB-NK) were also transduced, left 48h in cell culture and then used for cytotoxicity experiments. Histone silencing efficiency was measured by Western Blot with H2AZ, H4, acetylated H4 (Cell Signaling Technology) and H1.5 (Thermo Fisher Scientific) antibodies and secondary Anti-Rabbit IgG-HRP antibody (Cell Signaling Technology). For PB-NK Histone H2AZ silencing was measured by Western Blot and Flow cytometry with H2AZ antibody and adding either Anti-Rabbit IgG-HRP or Anti-Rabbit IgG-Alexa Fluor-647 as secondary antibody.

1. Martin-Antonio B, Najjar A, Robinson SN, Chew C, Li S, Yvon E, et al. Transmissible cytotoxicity of Multiple Myeloma cells by NK cells mediated by vesicle trafficking. Cell death and differentiation. 2015;22(1):96-107.
